# Experimental and Mathematical Model of Platelet Hemostasis Kinetics

**DOI:** 10.3390/cells14090677

**Published:** 2025-05-07

**Authors:** Bogdan Gerda, Anastasiya Volkova, Irina Dobrylko, Aleksandra Yu. Andreyeva, Thomas Dandekar, Mikhail A. Panteleev, Stepan Gambaryan, Igor Mindukshev

**Affiliations:** 1 Sechenov Institute of Evolutionary Physiology and Biochemistry, Russian Academy of Sciences, 44 Thorez Ave., 194223 Saint Petersburg, Russia; bgerda2525@gmail.com (B.G.); 10a.a.volkova@gmail.com (A.V.); dobrilko@mail.ru (I.D.); andreevaal@gmail.com (A.Y.A.); iv_mindukshev@mail.ru (I.M.); 2 A.O. Kovalevsky Institute of Biology of the Southern Seas, Russian Academy of Sciences, 14 Lenninsky Ave., 119991 Moscow, Russia; 3 Department of Bioinformatics, Biocenter, Am Hubland 1, D-97074 Würzburg, Germany; thomas.dandekar@uni-wuerzburg.de; 4 Center for Theoretical Problems of Physicochemical Pharmacology, Russian Academy of Sciences, 30 Srednyaya Kalitnikovskaya St., 109029 Moscow, Russia; mapanteleev@yandex.ru

**Keywords:** platelets, hemostasis, platelet phenotypes, signaling cascades, mathematical modeling, laser diffraction

## Abstract

Upon activation, platelets undergo rapid phenotypic transitions to maintain hemostasis, yet the kinetics governing these transitions remain poorly quantified. We present an integrated experimental and mathematical model describing platelet transitions between resting, activated, aggregating, inhibited, and exhausted phenotypes, determined by experiment rate constants for these reactions. Theoretical simulations of platelet transitions accurately describe the independently determined experimental read-out. Platelet aggregation under the conditions used directly correlates with the activation of αIIbβ3 integrins, demonstrating that the parameters of platelet aggregation achieved by the laser diffraction technique can be used for the evaluation of the rapid activation and deactivation kinetics of αIIbβ3 integrins. We demonstrate that platelet desensitization occurs at multiple activation stages, with distinct kinetic profiles for shape change and integrin deactivation. We also show that even 5 s of receptor-mediated PKA activation (iloprost) is sufficient for a complete inhibition of ADP-induced platelet aggregation. However, when iloprost was added after platelet stimulation by ADP, platelet activation was not fully inhibited, and after 180 s, aggregation became irreversible. The presented data help to understand the mechanisms of platelet transition between different phenotypes. The model effectively characterizes key physiological phenotypes and can serve as a modular framework for integration into more comprehensive models.

## 1. Introduction

In response to endothelial injury, platelets rapidly change shape and function, effectively adopting a new phenotype to maintain hemostasis. A series of key events drives platelet phenotype changes: (1) adhesion to the damaged endothelial layer, (2) initial activation leading to shape change, (3) granule release and activation of αIIbβ3 integrin receptors on the platelet surface, (4) platelet aggregation mediated by fibrinogen binding through αIIbβ3 receptors, (5) formation of procoagulant platelets contributing to thrombin generation, (6) platelet-driven coagulation resulting in thrombus formation stabilized by fibrin, and (7) programmed cell death followed by the release of microparticles [1,2,3,4,5]. In addition to activating reactions, inhibitory and reversible processes (such as disaggregation) are crucial for the localization of thrombus formation [6,7]. Heterogeneous subpopulations of platelets with activated and/or refractory phenotypes, which often actively contribute to the progression of inflammatory or oncological processes, have been described [8,9,10,11]. For example, distinct platelet subpopulations, described as “procoagulant”, “angry”, “coated”, “exhausted”, or “sticky”, have been identified in various pathophysiological conditions [12]. Exhausted platelets are of particular interest, as they arise from a sustained hyperactivation of circulating platelets and exhibit a markedly diminished or absent responsiveness to agonists [11]. Exhausted platelets are commonly observed under chronic stimulation in multiple disease states, including sepsis, large tumors, and stroke [8].

Several distinct terms to characterize platelet phenotypes have been introduced, including “reticulated”, “senescent” [13], “quiescent” or “resting” [14], “aggregating”, “secretory”, “procoagulant” [15], “coated” (COAT) [16], “primed” [17], “angry” [18], “sticky” [19], and “exhausted” [20]. However, despite the increasing diversity of the identified platelet phenotypes and their growing links to various pathological conditions, there is still no widely accepted classification.

A mathematical model that describes the kinetics of platelet transitions between distinct subpopulations in response to various stimuli could help to understand the mechanisms of platelet transition between different phenotypes. In several reviews, a variety of hemostasis models and the complex interactions between platelet adhesion, aggregation, and coagulation dynamics [21,22,23] are described. Models of platelet aggregation can be broadly categorized into two groups: mechanistic models [24,25] and models utilizing the Smoluchowski equation and mass action kinetics [26]. The latter group includes recently published models that, for the first time, characterize the dynamics of reversible platelet aggregation [26,27]. These models, however, do not incorporate the effects of cyclic nucleotide pathway activation on platelet disaggregation, even though this process may be essential for the regulation of thrombus structure [28,29]. Several studies have focused on modeling cAMP/cGMP signaling in platelets, providing insights into drug effects and the involvement of phosphodiesterases in the context of antiplatelet therapy [30,31]. The mentioned models also do not address the processes that characterized platelet disaggregation. Integrin αIIbβ3 activation is an essential step in platelet aggregation [11]. Patel and colleagues developed a detailed dynamic systems model describing the irreversible activation of integrin αIIbβ3 mediated by ADP [32]. However, in a recent review, it was proposed that agonist-induced αIIbβ3 activation is inherently a reversible process [33].

Thus, despite significant advances in mathematical modeling, current approaches do not fully capture the dynamics of changes between different platelet phenotypes. Here, using our well-established laser diffraction method of platelet activation analysis [34,35] controlled by flow cytometry analysis, we measured key dynamic parameters to develop a simple but powerful kinetic model of platelet transition. For the first time, we present a model of platelet hemostasis that describes the transition of platelets between different phenotypes (resting, activated, aggregating, inhibited, and exhausted phenotypes) and models of disaggregation from various activation states, both during activation by agonists (ADP, thrombin) and under parallel inhibition via the cAMP/cGMP signaling pathway. Furthermore, we show that integrin αIIbβ3 deactivation occurs only within the first few minutes following activation by ADP, which supports the presented view of an initially intermediate conformation of the integrins [36].

## 2. Materials and Methods

### 2.1. Ethics Approval

This study was conducted in accordance with the Declaration of Helsinki, and all experimental protocols were reviewed and approved by the Ethics Committee of the Sechenov Institute of Evolutionary Physiology and Biochemistry, RAS (protocol no. 03–02 from 28 February 2024). Signed informed consent was obtained from all participants prior to venipuncture.

### 2.2. Reagents and Working Buffers

Adenosine diphosphate (ADP), thrombin receptor activator peptide 6 (TRAP), sodium nitroprusside (SNP), iloprost (Ilo), and working buffer components (HEPES, NaCl, KCl, MgCl_2_, D-glucose, EGTA, CaCl_2_) were purchased from Sigma-Aldrich (Darmstadt, Germany). PAC-1-FITC were from BD Biosciences (Heidelberg, Germany). Fluo-3-AM was obtained from Invitrogen (Carlsbad, CA, USA). The following buffers were used: HEPES buffer with Ca^2+^ (150 mM NaCl, 5 mM KCl, 1 mM MgCl_2_, 10 mM D-glucose, 10 mM HEPES, pH 7.4, 2 mM CaCl_2_) and HEPES buffer with EGTA (150 mM NaCl, 5 mM KCl, 1 mM MgCl_2_, 10 mM D-glucose, 10 mM HEPES, pH 7.4, 2 mM EGTA). The osmolality of the buffers (300 mOsm/kg H_2_O) was controlled using a cryoscopic osmometer Osmomat 030 (Gonotec GmbH, Berlin, Germany).

### 2.3. Platelet Preparation

All donors were generally healthy women and men without pre-existing cardiovascular disease, aged between 18 and 40 years. Venous blood was collected from donors by caudal venipuncture in Citrate 9NC (0.106 mol/L/3.2%) S-monovette tubes (Sarstedt, Nümbrecht, Germany) with an addition of 2 mM EGTA and was centrifuged at 300× *g* (centrifuge ELMI-50CM, Elmi, Riga, Latvia) for 8 min at room temperature (RT). For the preparation of platelet-poor plasma (PPP), PRP was centrifuged at 10,000× *g* for 10 min at RT. Platelet count and parameters were monitored by the Medonic-M20 hematological counter (Boule Medical A.B., Stockholm, Sweden).

### 2.4. Flow Cytometry

The analysis was performed using a CytoFLEX flow cytometer (Beckman Coulter, Brea, CA, USA) at the Core Facility of the Institute of Evolutionary Physiology and Biochemistry, Russian Academy of Sciences. Each sample was assessed based on 15,000 events.

#### Analysis of Platelet Integrin αIIbβ3 Activation

The level of αIIbβ3 integrin activation was determined using PAC-1 antibodies conjugated with FITC, which specifically bind to activated αIIbβ3 integrins. PAC-1 antibodies (2 µL) were added to a 20 µL suspension of platelets (3 × 10^8^ cells/mL) and diluted in 400 µL of phosphate-buffered saline (PBS) containing 0.3% bovine serum albumin. Detection was performed using a 488 nm laser and fluorescence was measured at FL1 (525 nm).

### 2.5. Laser Diffraction Method

The laser diffraction method (laser microparticle analyzer, LaSca-TM, BioMedSystems Ltd., Saint Petersburg, Russia) was previously described in our earlier study [34,35,37]. Briefly, a 650 nm laser beam was passed through a platelet suspension under continuous stirring (1200 rpm, 37 °C) in a cuvette, and light scatter intensity (LSI) was recorded at various angles. The experimental model for investigating platelet hemostasis was established by diluting PRP in HEPES buffer to achieve a final platelet concentration of 2 × 10^7^ cells/mL.

#### 2.5.1. Analysis of Shape Change and Aggregation

The shape change reaction was induced by low doses of ADP (20–200 nM) and TRAP (100–1000 nM). The aggregation reaction was induced by higher doses of ADP (300–5000 nM) and TRAP (2–5 µM). Specific doses depended on the experimental setup and the platelet response. Platelet shape change was characterized by an increase in LSI at a scattering angle of 12°. Aggregation was defined by an increase in LSI at 1°, accompanied by a simultaneous decrease in LSI at 12°. The shape change reaction was evaluated based on the reaction rate calculated at 5 s from the process onset in LSI(12) ($V_{sh}$). Aggregation was assessed in LSI(1) by the area under the curve over 120 s (${AUC}_{agg}$), or the rate calculated at 20 s ($V_{agg}$). For a detailed description, see Supplementary Figure S1A,B.

#### 2.5.2. Analysis of Platelet Ca^2+^ Mobilization

Intracellular calcium [Ca^2+^]_i_ dynamics were assessed using the upgraded laser microparticle analyzer LaSca-TMF, equipped with a 488 nm laser and an FL1 fluorescence detector (527 nm) (BioMedSystems Ltd., Saint Petersburg, Russia), as detailed in [37]. Platelet-rich plasma (PRP) was incubated with Fluo-3 AM (final concentration: 5 µM) in the dark for 60 min at room temperature. Following incubation, the PRP was diluted with HEPES buffer. Changes in ([Ca^2+^]_i_) dynamics were monitored using the FL1 fluorescence detector (527 nm) and were analyzed as changes in the area under the fluorescence intensity curve over 20 s (${AUC}_{Ca}$) (see Supplementary Figure S1C).

#### 2.5.3. Desensitization of Platelets from the Shape Change State

Platelets were first weakly activated with a low concentration of ADP (100 nM), inducing only shape change. After a delay, a second stimulation with a high concentration of ADP (1000, 2000, 5000 nM) was applied. The decrease in the aggregation response was assessed in comparison to the control, where platelets were immediately activated with the corresponding high dose of ADP.

#### 2.5.4. Desensitization of Platelets from the State of Activated Integrins

To activate αIIbβ3 integrins, platelets were stimulated with a high concentration of ADP (1000, 2000, 5000 nM), and platelet interactions were halted by a temporary cessation of stirring to prevent further aggregation. The decrease in the aggregation response following the resumption of stirring was assessed in comparison to the control condition, where stirring was not interrupted. Since we established a correlation between integrin activation (PAC-1 binding) and the initial aggregation rate ($V_{agg}$) in Section 3.2.1, integrin activity was indirectly assessed using this parameter.

#### 2.5.5. Time-Dependent Inhibition of Platelet Activation by Cyclic Nucleotide Pathways

A saturating dose of iloprost (5 nM) or SNP (10 µM) was added to the platelet suspension at varying time intervals (ranging from 180 to 2 s) prior to activation with ADP (5000 nM for calcium dynamics and aggregation, 100 nM for shape change). Control experiments were conducted using the same ADP concentrations without iloprost or SNP.

#### 2.5.6. Platelet Disaggregation Mediated by Cyclic Nucleotide Pathways

A saturating concentration of iloprost (5 nM) or SNP (10 µM) was added at varying time points (5–180 s) following platelet activation with ADP (5000 nM). Disaggregation was assessed based on the reduction in LSI at 1° after reaching 100% aggregation and quantified as a percentage decrease in the signal from its maximum value (Disagg) (see Supplementary Figure S1A).

#### 2.5.7. Dose-Dependent Inhibition of Platelet Activation by Cyclic Nucleotide Pathways

Platelet activation was suppressed using varying concentrations of iloprost (ranging from 0.025 to 0.3 nM for the shape change reaction and from 0.1 to 2 nM for aggregation). After 60 s, platelets were stimulated with ADP (100 nM for shape change and 5000 nM for aggregation). Control experiments were conducted using platelets activated with the corresponding ADP concentrations in the absence of iloprost.

### 2.6. Data Processing and Analysis

Data are presented as mean ± SD; *n* is sample size. Dose–response relationships were evaluated based on the half-maximal effective concentration of the agonist/antagonist (${EC}_{50}$/${IC}_{50}$) and the Hill coefficient (h). Statistical analysis of pH and fibrinogen effects was carried out using one-way ANOVA followed by Dunnett’s test, with statistical significance set at *p* < 0.05. Flow cytometry data were analyzed using CytExpert software v2.4 (Beckman Coulter, Brea, CA, USA), and laser diffraction data were processed using the original software LaSca_32v.1750 (BioMedSystems Ltd., Saint Petersburg, Russia) of the laser particle analyzer LaSca-TM. Primary data analysis was performed using Python 3.12.0 with the following libraries: Pandas 2.2.3, SciPy 1.15.2, Matplotlib 3.10.0, and NumPy 2.2.4. Systems of ordinary differential equations (ODEs) were solved using the LSODA method (Livermore Solver for Ordinary Differential Equations) from the SciPy library. Global optimization was conducted via the basin-hopping (BH) algorithm, and local optimization was performed using the L-BFGS (limited-memory Broyden–Fletcher–Goldfarb–Shanno) method. Model fit accuracy was evaluated using the coefficient of determination (R^2^).

## 3. Results

### 3.1. Kinetic Model of Platelet Hemostasis

#### 3.1.1. Schematic Representation of the Model

**Figure 1:**
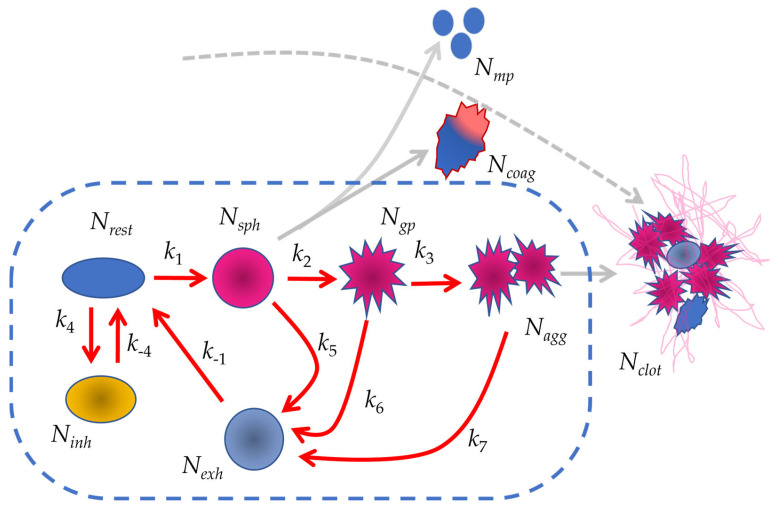
Kinetic scheme of platelet hemostasis with distinct cellular phenotypes. The phenotypes considered in the model include the following: “rest”—resting, non-activated platelets; “inh”—inhibited platelets; “sph”—sphered platelets, formed during the shape change reaction; “gp”—platelets with activated αIIbβ3 integrins; “agg”—aggregated platelets, where platelet aggregates are stabilized via fibrinogen bridges between integrins; “exh”—refractory (exhausted) platelets, which arise from reversible processes such as disaggregation and remain temporarily unresponsive to further activation. The kinetic parameters governing these transitions are as follows: $k_{1}$ is the rate constant for the transition from resting platelets ($N_{rest}$) to sphered platelets ($N_{sph}$); $k_{2}$ is the rate constant for the transition from sphered platelets ($N_{sph}$) to integrin-activated platelets ($N_{gp}$); $k_{3}$ is the rate constant for the transition from integrin-activated platelets ($N_{gp}$) to aggregated platelets ($N_{agg}$); $k_{4}$ is the rate constant for the transition from resting platelets ($N_{rest}$) to inhibited platelets ($N_{inh}$); $k_{-4}$ is the rate constant for the reversal of inhibition, transitioning inhibited platelets ($N_{inh}$) back to the resting state ($N_{rest}$); $k_{5}$ is the rate constant for the transition from sphered platelets ($N_{sph}$) to the refractory state ($N_{exh}$); $k_{6}$ is the rate constant for the transition from integrin-activated platelets ($N_{gp}$) to the refractory state ($N_{exh}$); $k_{7}$ is the rate constant for the transition from aggregated platelets ($N_{agg}$) to the refractory state ($N_{exh}$); ($N_{mp}$) microparticles; ($N_{coag}$) procoagulant platelets; ($N_{clot}$) clot formation. The section of the platelet hemostasis pathway analyzed in the present model is outlined in blue and indicated with red arrows.

During activation, platelets undergo a series of morphological and functional transformations, which are characterized by the transition of different phenotypes. By using our experimental conditions, we proposed six primary platelet phenotypes and describe their interconversions within a kinetic model (Figure 1). Resting platelets (rest) are quiescent, non-activated platelets in their native state. Sphered platelets (sph) represent an early activation stage characterized by shape change. Thrombotic platelets (gp) exhibit activated αIIbβ3 integrins, enabling adhesion. Aggregating platelets (agg) form platelet–platelet aggregates via fibrinogen bridging. Inhibited platelets (inh) remain functionally suppressed due to activation of cAMP/cGMP signaling pathways. Exhausted platelets (exh) are refractory to further activation for a defined period, representing a desensitized or depleted state. The presented scheme of platelet transformation will be used for the subsequent analysis and for the classification of different phenotypes.

#### 3.1.2. The Mathematical Framework of the Model

The transitions between different phenotypes were characterized using chemical kinetic approaches, which are frequently applied for modeling signaling cascades [32,38]. The reactions were treated as first-order processes, except for the aggregation reaction. The rate constants for these reactions were either directly calculated from experimental data or estimated by fitting theoretical curves to experimental data. Initially, the model assumes that 100% of the platelets are in the “rest” phenotype. The dynamics of the phenotypic distribution were computed using a system of six differential Equations (1).

(1) $$\left\{\begin{array}{l} dN_{rest}/dt=k_{-4}N_{inh}+k_{-1}N_{exh}-k_{4}N_{rest}-k_{1}N_{rest}\\ dN_{sph}/dt=k_{1}N_{rest}-k_{2}N_{sph}-k_{5}N_{sph}\\ dN_{gp}/dt=k_{2}N_{sph}-{k_{3}N_{gp}}^{2}-k_{6}N_{gp}\\ dN_{agg}/dt={k_{3}N_{gp}}^{2}-k_{7}N_{agg}\\ dN_{exh}/dt=k_{5}N_{sph}+k_{6}N_{gp}+k_{7}N_{agg}-k_{-1}N_{exh}\\ dN_{inh}/dt=k_{4}N_{rest}-k_{-4}N_{inh} \end{array}\right.,$$

where N represents the percentage of a given phenotype (subpopulation) in the total platelet population (%), and k is the first-order rate constant (1/s), with the exception of the aggregation reaction, where $k_{3}$ is the second-order rate constant.

The system of six differential equations is further augmented by the dependencies of the rate constants on the concentrations of agonists interacting with platelets via receptors. The phenotypic changes in the cells are driven by the activation of intracellular signaling pathways [12]. According to [39,40,41], the relationship between the agonist concentration and the cellular response in intracellular signaling cascades (e.g., the MAP kinase pathway) is well approximated by the Hill equation; therefore, we used this equation. Equation (2) is as follows:

(2) $$\frac{dOutput}{dt}={Output}_{max}\frac{{Input}^{h}}{K_{0.5}^{h}+{Input}^{h}},$$

where Output denotes the system’s response to the stimulus, ${Output}_{max}$ is the maximum response, Input represents the stimulus, $K_{0.5}$ is the stimulus at which half of the maximum response is reached (${EC}_{50}$), and h is the Hill coefficient.

For the dose–response relationship of the rate constant as a function of the agonist, the Hill equation takes the following form (Equation (3)):

(3) $$k=k_{max}\frac{{\left[A\right]}^{h}}{{EC}_{50}^{h}+{\left[A\right]}^{h}},$$

where k is the reaction rate constant, $k_{max}$ is its maximum value at a saturating agonist dose, $\left[A\right]$ is the agonist concentration, and ${EC}_{50}$ is the agonist concentration that produces half of the maximum response.

The inhibitory dose-dependent effect (a stimulus that suppresses the reaction) can also be described using a modified Hill formalism, and the equation takes the following form (Equation (4)):

(4) $$k=k_{max}\frac{{IC}_{50}^{h}}{{IC}_{50}^{h}+{\left[I\right]}^{h}},$$

where ${IC}_{50}$ is the inhibitor concentration required to achieve half-maximal inhibition of the reaction and [I] is the inhibitor concentration.

A more detailed description of the mathematical model of platelet hemostasis, incorporating the complex interactions of concurrent activating and inhibitory stimuli, is provided in Supplementary Section S2 (Equations (S1)–(S8)). The subsequent experimental section is dedicated to quantifying reaction rate constants, identifying factors that modulate these reactions, and establishing dose–response relationships for both activatory and inhibitory stimuli.

### 3.2. Experimental Parameters for Hemostasis Kinetics Analysis

Platelet aggregation depends on calcium, fibrinogen, and cell concentrations in the buffer. Therefore, we first established the optimal concentrations of these parameters for the subsequent experiments.

#### 3.2.1. Identification of Optimal Platelet Concentration for the Laser Diffraction Method

All our experiments are performed in diluted PRP; therefore, it is important to determine the optimal dilution range in which platelets exhibit a measurable aggregation response, suitable for quantitative analysis by laser diffraction.

**Figure 2:**
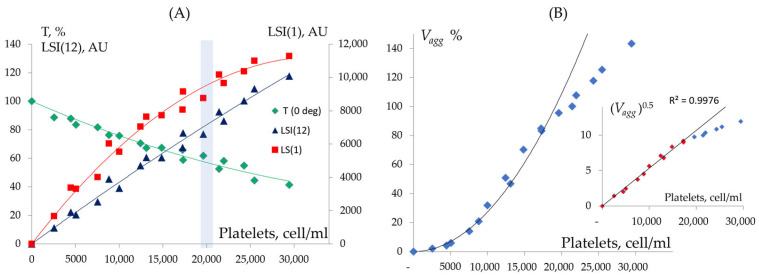
Dependence of light scattering intensity and aggregation on platelet concentration. (**A**) Transmission values (T(0°)) and light scattering intensities at 1° (LSI(1)) and 12° (LSI(12)). The cuvette background is used as the 100% transmission reference. (**B**) The initial aggregation rate ($V_{agg}$), assessed by the dynamics of LSI(1) at 20 s after the addition of 5 μM ADP. In this set of experiments, 100% $V_{agg}$ corresponds to the value observed at a platelet concentration of 20,000 cells/μL. The data were obtained using the LaSca analyzer with a red laser (λ = 650 nm).

The laser diffraction method is employed when PRP is diluted 20–30 times. This dilution is necessary due to the requirements of the laser diffraction technique, which allows for accurate measurements of light scattering intensity only under conditions of single scattering. Single scattering occurs when there is a linear relationship between the light scattering intensity (LSI) and cell concentration, and/or when the transmission signal (T) remains above 50%. These conditions are reached at platelet concentrations of up to 22,000 cells/μL (Figure 2A).

Platelet aggregation occurs through stochastic collisions and follows second-order formal reaction kinetics, where the reaction rate is governed by the collision frequency and the effectiveness of these collision, as described by the Smoluchowski equation [26,42]. Indeed, at platelet concentrations below 15,000 cells/µL, aggregation kinetics follow a quadratic dependence, $V_{agg}\;\sim \;N^{2}$ (Figure 2B). However, at concentrations exceeding 18,000 cells/µL, the aggregation rate exhibits a linear dependence on the platelet concentration, deviating from the Smoluchowski equation, indicating that under our experimental conditions, collision frequency is no longer the limiting factor for platelet aggregation. Therefore, for all our experiments, the platelet concentration was kept between 18,000 and 22,000 cells/µL.

#### 3.2.2. Aggregation Is Dependent on the Calcium Concentration in the Medium

**Figure 3:**
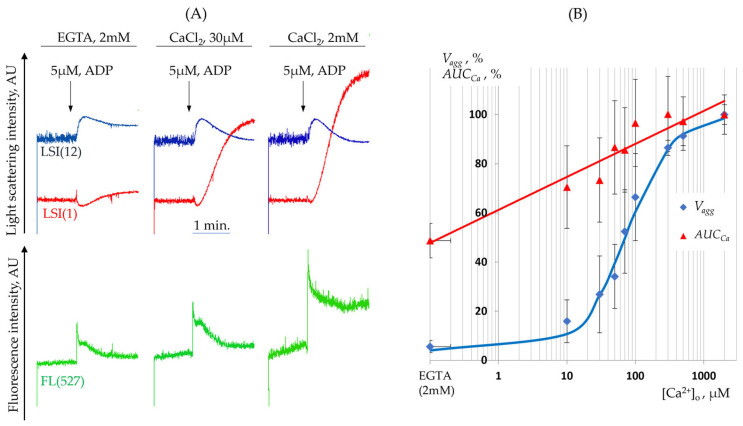
Dependence of platelet responses on extracellular calcium ion concentration, [Ca^2+^]_o_. (**A**) Representative experimental traces of LSI(1), LSI(12), and FL(527) (excitation at 488 nm, emission at 527 nm), illustrating platelet shape change and intracellular calcium dynamics (Cai) at varying calcium concentrations in the medium. Platelets were stimulated with 5 µM ADP at 37 °C. (**B**) Dependence of the initial aggregation rate ($V_{agg}$), measured from LSI(1) dynamics at 20 s post-ADP addition, and the increase in [Ca^2+^]_i_ (quantified by ${AUC}_{Ca}$—the area under the curve of the calcium signal over 20 s, measured by FL (527) fluorescence). In each experimental series, the $V_{agg}$ and ${AUC}_{Ca}$ values at [Ca^2+^]_o_ = 2 mM and [ADP] = 5 µM were set as 100%. Data are presented for eight experimental replicates.

Given the crucial role of extracellular calcium ions ([Ca^2+^]_o_) in the aggregation process [43], we investigated the relationship between [Ca^2+^]_o_ and platelet aggregation to determine the optimal calcium concentration in the medium. The shape change reaction is independent of extracellular calcium (Figure 3A), whereas the aggregation is strictly dependent on [Ca^2+^]_o_ and does not occur in calcium-free conditions. The half-maximal effective concentration of calcium in the external medium (${EC}_{50}$), which elicits 50% of the aggregation response, was 76 ± 5 µM [Ca^2+^]_o_. Maximum levels of [Ca^2+^]_i_ and aggregation are achieved at the physiological calcium concentration (2 mM); therefore, in all subsequent experiments, this [Ca^2+^]_o_ concentration was used.

#### 3.2.3. Aggregation Is Not Affected by pH Within Physiological Ranges

We found no data on the effect of pH on aggregation and therefore investigated whether aggregation could be dependent on pH in the buffer. The results, presented in Supplementary Figure S2, indicate that within the physiological pH range (7.0–7.8), pH does not affect the aggregation response induced by ADP or TRAP; therefore, in the media, pH was adjusted to 7.4 in all experiments.

#### 3.2.4. Fibrinogen Concentration Is Not a Limiting Factor of Platelet Aggregation

Aggregation is determined by the binding of integrins to fibrinogen; therefore, we tested whether the fibrinogen levels in our diluted PRP conditions were sufficient to achieve maximal aggregation. To increase fibrinogen concentration, different amounts of PPP (50 or 100 µL) were added to the buffer with PRP (platelet concentration was maintained constant). The addition of PPP (increase in the fibrinogen concentration) had no effect on the aggregation response (Supplementary Figure S3), indicating that fibrinogen concentration is not a limiting factor for aggregation under our experimental conditions.

In summary, the presented results establish the optimal experimental conditions for studying platelet hemostasis kinetics using the low-angle light scattering method. Briefly, blood is stabilized with citrate containing 2 mM EGTA. Before starting all experiments, platelets are maintained in PRP at room temperature. Immediately prior to measurements, PRP is diluted 20- to 30-fold in a buffer with 2 mM [Ca^2+^]_o_, pH 7.4, and the final platelet concentration is in the range of ~20,000 cells/µL.

### 3.3. Rate Constants of Key Hemostatic Reactions and Dose–Response Relationships

#### 3.3.1. Quantitative Characterization of Shape Change and Aggregation Reactions

Determination of $k_{1}$ Constant (Shape Change Reaction)

**Figure 4:**
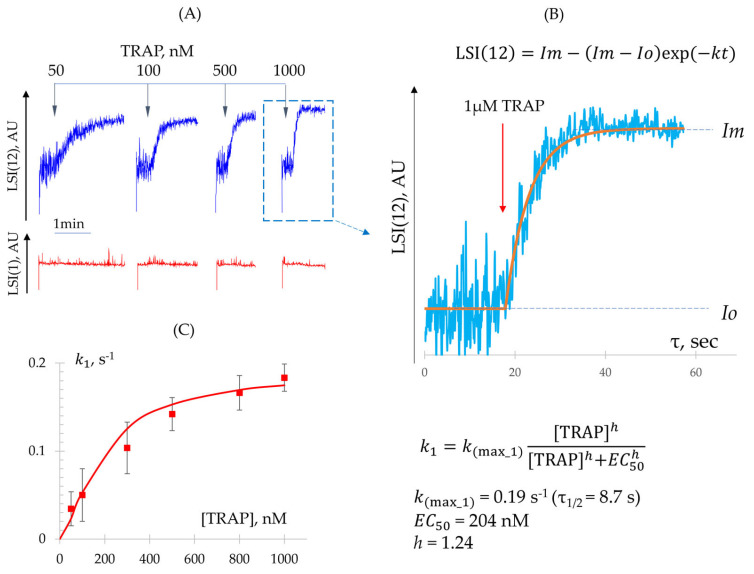
The dynamics of the shape change reaction. (**A**) Experimental dependence of aggregation (LSI(1)) and shape change (LSI(12)) reactions on low concentrations of the agonist (TRAP). (**B**) Determination of $k_{\left(max\_1\right)}$ from the kinetics of the shape change response at a saturating dose of the agonist (TRAP). $I_{m}$ denotes the maximum light scatter intensity (arbitrary unit, AU); $I_{o}$ is the baseline light scatter intensity corresponding to non-activated cells (AU). (**C**) The rate constant for shape change ($k_{1}$) as a function of agonist concentration (TRAP). The equation for calculating the rate constants, along with the values of the maximum rate constant and dose–response parameters, are also provided. AU—arbitrary units.

Low agonist concentrations induce a rapid shape change response without initiating platelet aggregation. To quantitatively characterize this process, we analyzed the kinetics of LSI(12) elevation following platelet activation with low doses of ADP and TRAP (Figure 4A), and determined ${EC}_{50}$ values (Table 1). The maximum rate constant, corresponding to the saturating dose of the agonist, was determined from experimental data (Figure 4B) using the following Equation (5):

(5) $$LSI\left(12\right)=I_{m}-\left(I_{m}-I_{o}\right)exp(-k_{max}t),$$

where $LSI\left(12\right)$ represents the experimental light scatter signal (arbitrary unit, AU); $I_{m}$ denotes the maximum light scatter intensity (AU); $I_{o}$ is the baseline light scatter intensity corresponding to non-activated cells (AU); $k_{max}$ is the rate constant of the reaction at the saturating agonist dose (1/s); and t is time in seconds (s).

Accordingly, the maximum rate constant $k_{\left(max\_1\right)}$, corresponding to the saturating concentration of the agonist, was 0.19 ± 0.05 1/s (n = 23). Additionally, rate constants for other agonist concentrations were also calculated using Equation (5) and compared with values derived from Equation (3) (see Section 3.1.2), using the determined $k_{\left(max\_1\right)}$, ${EC}_{50}$, and h values. The theoretically calculated and experimental data are directly correlated (Figure 4C).

Determination of $k_{2}$ − $k_{3}$ Constants (Integrin Activation and Platelet Aggregation)

**Figure 5:**
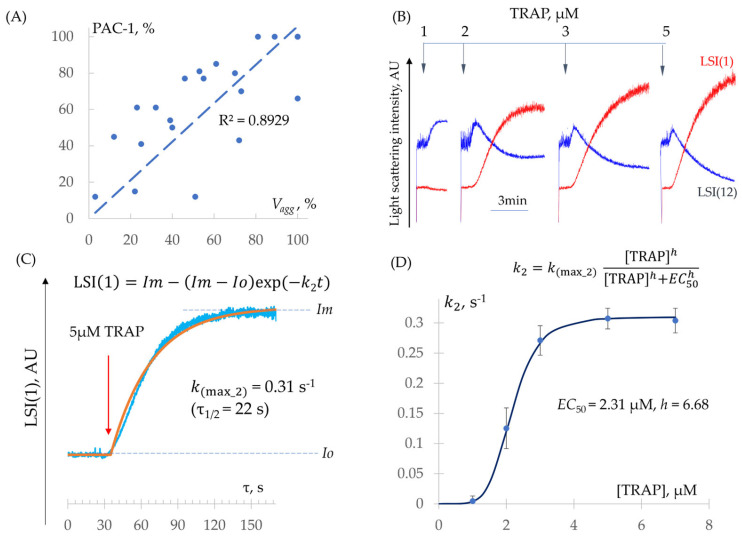
The dynamics of the aggregation (integrin activation) reaction. (**A**) Correlation between αIIbβ3 integrin activation, quantified by flow cytometry using FITC-conjugated PAC-1 antibodies, and the initial aggregation rate, assessed by laser diffraction. (**B**) Experimental dependence of aggregation (LSI(1)) and shape change (LSI(12)) on high agonist concentrations (TRAP). (**C**) Determination of $k_{\left(max\_2\right)}$ from the kinetics of the aggregation response at a saturating agonist dose (TRAP). $I_{m}$ denotes the maximum light scatter intensity (AU); $I_{o}$ is the baseline light scatter intensity corresponding to non-activated cells (AU). (**D**) The rate constant for αIIbβ3 integrin activation ($k_{2}$) as a function of agonist concentration (TRAP). The equation for calculating the rate constants, along with the values of the maximum rate constant and dose–response parameters, are also provided.

First, to confirm that platelet aggregation measured by the laser analyzer ($V_{agg}$) correlates with integrin αIIbβ3 activation, we used the flow cytometry method of integrin activation (PAC-1 antibodies) and showed a direct correlation (R^2^ = 0.89) between both methods (Figure 5A). Second, we demonstrated that at cell concentrations exceeding 18,000 cells/µL in the $k_{2}$ − $k_{3}$ phase, the $k_{3}$ reaction is not rate-limiting (see Section 3.2.1). Based on this, the limiting step is the $k_{2}$ reaction, meaning that the rate of aggregation is predominantly determined by the rate of integrin αIIbβ3 activation. Consequently, under our experimental conditions, aggregation measured by the laser analyzer allows for the quantitative assessment of integrin activation. The subsequent aggregation rate ($k_{\left(max\_3\right)}$) was set to be twice as high as $k_{\left(max\_2\right)}$ to ensure that this process did not become rate-limiting under the model conditions.

To quantitatively assess the dose dependence of integrin αIIbβ3 activation on the agonist, the reaction was induced using various concentrations of ADP and TRAP, and ${EC}_{50}$ values were then calculated from the elevation of LSI(1) (Figure 5B, Table 2). The maximum rate constant ($k_{\left(max\_2\right)}$) was determined using Equation (5) at a saturating concentration of TRAP: $k_{\left(max\_2\right)}$ = 0.03 ± 0.006 s^−1^ (n = 14) (Figure 5C). Furthermore, rate constants ($k_{2}$) at different TRAP concentrations were estimated using Equation (5) and compared with theoretical values calculated from Equation (3) (see Section 3.1.2) using determined $k_{\left(max\_2\right)}$, ${EC}_{50}$, and h. The theoretical and experimental dependencies closely matched (Figure 5D).

Thus, we demonstrated that under our experimental conditions, aggregation is limited by the rate of integrin αIIbβ3 activation and directly correlates with it, enabling a quantitative assessment of this process. We determined $k_{\left(max\_2\right)}$ (see Figure 1) along with the corresponding ${EC}_{50}$ and h values for ADP and TRAP in the integrin activation reaction. Additionally, we quantitatively characterized the shape change reaction, identifying $k_{\left(max\_1\right)}$ (see Figure 1), as well as ${EC}_{50}$ and h, for ADP and TRAP. To ensure that the aggregation reaction was not rate-limiting and always proceeded faster than integrin activation under the simulation conditions, we set $k_{\left(max\_3\right)}$ to be twice the value of $k_{\left(max\_2\right)}$.

#### 3.3.2. Quantitative Characterization of Platelet Desensitization Reactions

Platelets can return to a refractory state at different stages of activation, including shape change, integrin αIIbβ3 activation, and after aggregate formation during the disaggregation process [33]. The experiments described below were performed to quantitatively characterize the kinetics of these transitions.

Determination of $k_{5}$ (Desensitization from Shape Change State)

**Figure 6:**
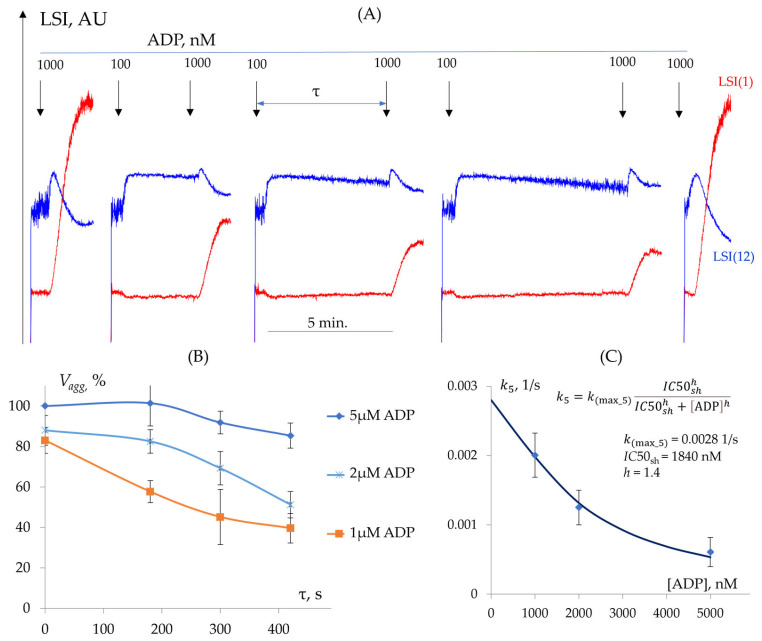
Desensitization of platelets from the shape change state. (**A**) Experimental dependence of aggregation responses (LSI(1), red) and shape change (LSI(12), blue) on the duration of the delay before the reactivation of the cells. (**B**) Relationship between the initial aggregation rate ($V_{agg}$) and the duration of the delay before reactivation for different ADP concentrations. (**C**) Dependence of the desensitization rate constant for shape change ($k_{5}$) on ADP concentration. The equation for calculating the rate constants, along with the values of the maximum rate constant and dose–response parameters, is also provided.

In our experimental conditions, the platelet shape change reaction is constant at least during 10 min of incubation with a low dose of agonists. However, it is not clear whether the stable shape change reaction could influence the next activation with a high dose of agonist. To assess this, we first induced a shape change reaction by 100 nM of ADP, and after a fixed time, we stimulated the platelets with higher (1, 2, 5 µM) concentrations and analyzed the aggregation response (Figure 6A). Platelet aggregation gradually decreased, with an elevated time between the second addition of ADP, and was dependent on the concentration of ADP (Figure 6B,C). These data were used for the calculation of time constants ($k_{5}$) (See Figure 1) (Figure 6B,C), the half-maximal inhibitory concentration (${IC}_{50}$ = 1840), and the Hill coefficient (h = 1.4) (Table 3). The maximum rate constant was derived by fitting the experimental dependence of $k_{5}$ on ADP concentration using Equation (4) (See Section 3.1.2), yielding $k_{\left(max\_5\right)}$ = 0.0028 1/s (Figure 6C).

Determination of $k_{6}$ (Desensitization from Integrin Activation State)

**Figure 7:**
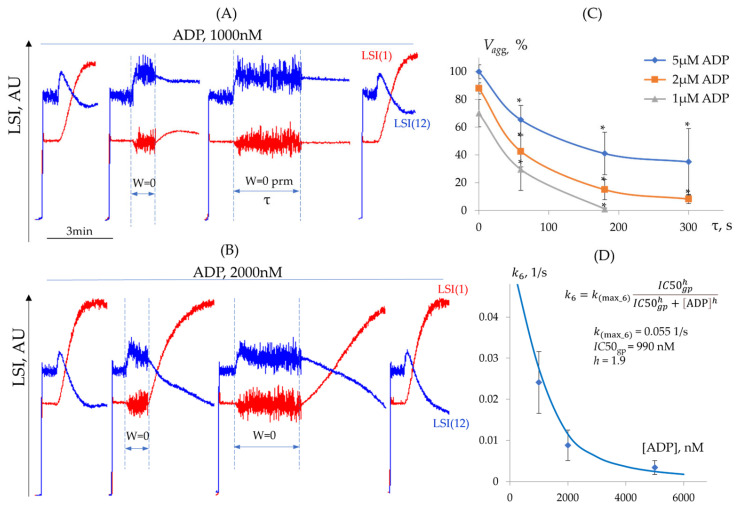
The desensitization of platelets from the activated αIIbβ3 integrin state. (**A**) Experimental dependence of aggregation (LSI(1), red) and shape change (LSI(12), blue) on the duration of the delay before reinitiating stirring following the addition of 1000 nM ADP. (**B**) Experimental dependence of aggregation (LSI(1), red) and shape change (LSI(12), blue) on delay duration before reinitiating stirring following the addition of 2000 nM ADP. (**C**) Dependence of the initial aggregation rate ($V_{agg}$) on delay duration before stirring resumption for different ADP concentrations. (**D**) Dependence of the rate constant $k_{6}$ for platelet desensitization from the activated αIIbβ3 integrin state on ADP concentration. The equation for calculating the rate constants, along with the values of the maximum rate constant and dose–response parameters, is also provided.

As was shown above (See Section 3.3.1) in our experimental conditions, aggregation directly correlated with integrin αIIbβ3 activation, which is consistent with the previous publication [33]. However, it is not clear whether already activated αIIbβ3 integrins could be inactivated when the collision of platelets is interrupted. Platelets were activated by different ADP concentrations to activate αIIbβ3 integrin; then, the stirrer was turned off to prevent cell interactions and aggregation. The stirrer was then switched on, and changes in aggregation were assessed in comparison to the control. Aggregation progressively declined with increasing stir interruption duration, indicating that the prevention of platelet collisions leads to the formation of exhausted platelets (Figure 7A,B). At low ADP concentrations, aggregation was completely abolished when stirring was delayed for more than 180 s before resumption (Figure 7A,C), whereas at high ADP concentrations, aggregation declined with prolonged delays but remained detectable even after 300 s (Figure 7B,C). Based on these experiments, we can assume that the prevention of collisions between platelets could return the αIIbβ3 integrins to an inactive or intermediate state, in which aggregation is reduced or absent. Moreover, the extent of this transition is directly dependent on the time of collisions prevention and ADP concentration.

These data allow for the calculation of the rate constants $k_{6}$ (See Figure 1) (Table 4) and ${IC}_{50}$= 990 nM and h = 1.9. Additionally, the value of the maximum rate constant was calculated by fitting the experimental dependence of $k_{6}$ on ADP concentration using Equation (4) (See Section 3.1.2), yielding $k_{\left(max\_6\right)}$ = 0.055 1/s (Figure 7D).

Thus, we demonstrated that incomplete platelet activation leads to platelet exhaustion and results in absent or reduced aggregation in response to agonist stimulation. We present data indicating that platelets could be desensitized from both the shape change and activated αIIbβ3 integrin states. We determined the maximum rate constants for these transitions ($k_{\left(max\_5\right)}$ and $k_{\left(max\_6\right)}$, respectively) and identified the dose–response parameters (${IC}_{50}$ and h) for these processes in relation to ADP concentration.

#### 3.3.3. Determination of $k_{4}$ (Quantitative Characterization of Platelet Inhibition Reactions)

Cyclic nucleotides (cAMP and cGMP) are among the major inhibitory signaling molecules in platelets [11,44,45]. Activation of cyclic nucleotide pathways not only suppresses platelet activation but also promotes disaggregation [29]; however, a quantitative characterization of these processes has not been provided.

**Figure 8:**
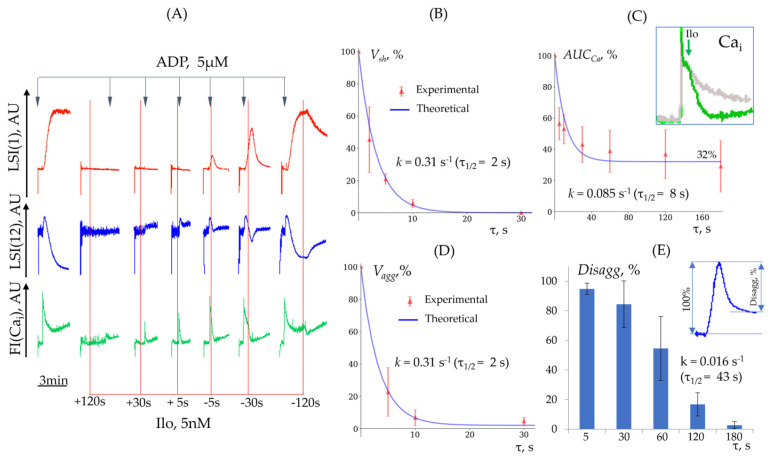
Inhibition of platelet activation by iloprost. (**A**) Temporal profiles of platelet aggregation (LSI(1), red), shape change (LSI(12), blue), and intracellular calcium dynamics (green) before and after the administration of iloprost are displayed. (**B**) Rate of shape change reaction ($V_{sh}$) versus time between iloprost addition and ADP-induced activation. (**C**) Intracellular calcium dynamics (${AUC}_{Ca}$) versus time between iloprost addition and ADP-induced activation. In the upper right corner, calcium efflux from the cytoplasm is shown upon the addition of iloprost after platelet activation (green), compared to the control condition without iloprost (gray). (**D**) Initial aggregation rate ($V_{agg}$) versus time between iloprost addition and ADP-induced activation. (**E**) Disaggregation (Disagg) versus time between initial activation and iloprost addition. $V_{sh}$, $V_{agg}$, and ${AUC}_{Ca}$ are normalized to control experiments without the inhibitor (100%).

For the quantitative characterization of the inhibition and disaggregation processes induced by cAMP and cGMP, we conducted two sets of experiments. First, we evaluated the dependence of platelet activation from the time of cyclic nucleotide activation (Figure 8A). For this, the activators of the cAMP pathway (iloprost) and cGMP pathway (SNP) were added prior to or after platelet activation by high ADP (5 µM) concentration. The shape change reaction was completely prevented only when iloprost (5 nM) was added 120 s before ADP, whereas starting from a 30 s delay to ADP addition, this reaction was already detectable and was not inhibited in subsequent conditions (Figure 8A, middle panel). Even 5–10 s of iloprost addition before ADP was sufficient for a complete suppression of platelet aggregation (Figure 8A upper panel, D). The calcium response declined more gradually, remaining pronounced at 30 s and not reaching zero even after 180 s, instead stabilizing at 30–40% of the control signal (Figure 8A lower panel, C). Because the shape change reaction reaches its maximum values at 100 nm of ADP concentration, we quantified it for this ADP concentration (Figure 8B, Supplementary Figure S4). The inhibition rate constants for shape change, aggregation, and the suppression of [Ca^2+^]_i_ dynamics for iloprost are presented in Table 5.

Then, we evaluated whether the activation of cyclic nucleotide pathways could induce disaggregation reaction (see Section 2.5.6 and Figure 7A). In these experiments, iloprost was added 5, 30, and 120 s after ADP (5 nM). Iloprost induced the disaggregation process depending on the time of its addition. When administered 5 s after activation, iloprost induced complete disaggregation; after 60 s, disaggregation was ~50%, and no disaggregation was observed after 3 min (Figure 8E), indicating that activation of the cAMP pathway induces disaggregation only within a narrow time window, lasting no more than 3 min.

**Figure 9:**
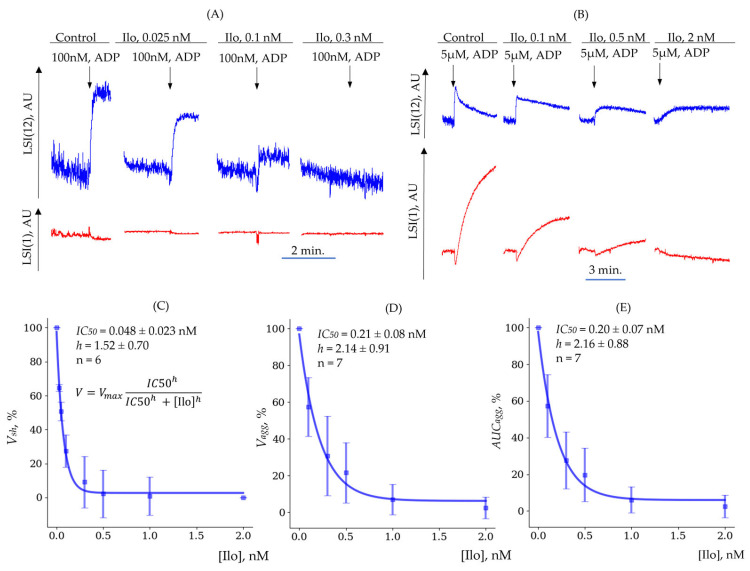
Effect of iloprost (Ilo) on shape change and aggregation responses. (**A**) Experimental dependence of aggregation (LSI(1), red) and shape change (LSI(12), blue) on iloprost concentration with weak ADP activation (100 nM). (**B**) Experimental dependence of aggregation (LSI(1), red) and shape change (LSI(12), blue) on iloprost concentration with strong ADP activation (5 µM). (**C**) Experimental and theoretical dependencies of shape change rate ($V_{sh}$) on iloprost concentration [Ilo]. (**D**) Experimental and theoretical dependencies of aggregation rate ($V_{agg}$) on iloprost concentration [Ilo]. (**E**) Experimental and theoretical dependencies area under the aggregation curve (${AUC}_{agg}$) on iloprost concentration [Ilo]. Error bars represent standard deviation (sd). Theoretical curves are based on the Hill equation.

In the next set of experiments, we analyzed the dose–response parameters of iloprost for the shape change and aggregation reactions (Figure 9A,B). The calculated values of ${IC}_{50}$ and the Hill coefficient (h) are presented in (Table 6). Iloprost started to suppress the shape change response induced by 100 nM of ADP (Figure 9A,C), even at a very low concentration (0.025 nM), whereas at 5 µM of ADP, much higher concentrations of iloprost were required for the inhibition of the shape change and aggregation reactions (Figure 9B,D,E).

Thus, based on the presented data, we determined the time required for cAMP/cGMP activators to suppress platelet activation responses and calculated the corresponding reaction rate constants. We quantified the dose-dependent effects of iloprost on platelet activation. Furthermore, we demonstrated that the activation of cyclic nucleotide systems acts not only on resting platelets but also on already activated cells; however, it does so for a strongly limited time, lasting ~180 s after activation. All data concerning SNP’s effect on platelets are presented in Supplementary Section S6 (Supplementary Figure S5 and Supplementary Table S1).

### 3.4. Experimental and Theoretical (Modeled) Data Are Closely Related

The maximum rate constants ($k_{max}$), as well as the ${EC}_{50}$, ${IC}_{50}$, and h parameters, were experimentally determined for most reactions and are presented in the previous sections; however, some parameters are challenging to determine experimentally (e.g., $k_{-4}$, $k_{-1}$ and $k_{7}$; see Figure 1). These unknown parameters were estimated by fitting the theoretical curves to the experimental data, and the model accuracy was subsequently evaluated (Supplementary Figures S6–S9). Shifting the fitted parameters in either direction away from the optimal values leads to a discrepancy between the model predictions and the experimental data (Supplementary Figures S6 and S7). A summary table of all parameter values used in the modeling and the detailed algorithm for the calculation of all constants, accounting for variable agonist/antagonist concentrations, are presented in the Supplementary Material (Table S2 and Equations (S9)–(S57)).

**Figure 10:**
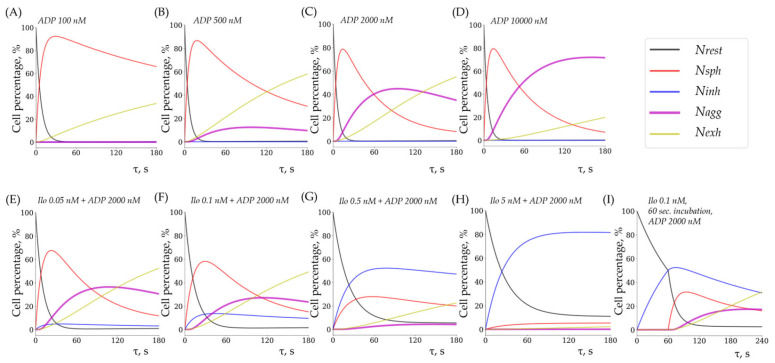
Simulated dose–response relationships for ADP and iloprost. (**A**) Exposure to 100 nM ADP only induces shape change in platelets, which subsequently transition into an exhausted phenotype. (**B**) A 500 nM dose of ADP results in weak platelet aggregation followed by exhaustion. (**C**) 2000 nM ADP leads to strong aggregation; however, rapid formation of exhausted cells and disaggregation are also observed. (**D**) A 10,000 nM dose of ADP induces stable, strong aggregation with slow exhaustion. (**E**) Treatment with 0.05 nM Ilo weakly suppresses aggregation upon simultaneous activation with 2000 nM ADP. Inhibited platelets are barely formed. (**F**) Increasing Ilo concentration to 0.1 nM raises the proportion of inhibited platelets, yet the aggregation response remains pronounced. (**G**) A 0.5 nM dose of Ilo nearly completely suppresses aggregation, with approximately half of the cells exhibiting an inhibited phenotype, though the shape change response remains evident. (**H**) A 5 nM dose of Ilo fully suppresses aggregation, yet a weak shape change response persists. (**I**) Pre-incubating platelets with Ilo for 60 s results in a stronger suppression of aggregation compared to the co-administration of Ilo and ADP at the same concentration (see panel (**F**)).

Here, for illustration, we present the results of the final model, which incorporates reverse reactions and non-competitive inhibition of forward reactions. Aggregation is absent at low ADP doses but increases with higher concentrations (Figure 10A–D). At moderately high ADP doses, pronounced disaggregation and the formation of exhausted platelets are observed (Figure 10C). At very high ADP concentrations, disaggregation is nearly absent within the studied time frame, and platelet exhaustion occurs slowly (Figure 10D). Iloprost treatment induces a dose-dependent suppression of aggregation (Figure 10E–H); however, shape change is not completely suppressed, even at high ADP concentrations (Figure 10H; see also Supplementary Figure S8). Pre-incubation of platelets with iloprost for 60 s leads to a stronger inhibition of aggregation compared to the simultaneous administration of iloprost and ADP (Figure 10F,I).

In summary, an in silico model was developed based on Hill’s formalism and chemical kinetics to characterize the phenotypic transformations of platelets during the early stages of hemostasis. The model incorporates activation reactions (shape change, integrin activation, aggregation), as well as inhibition and disaggregation (particularly induced by cAMP/cGMP activation). Key time constants and dose–response parameters for these reactions were accurately determined in experiments (see Figure 1), and the theoretical simulations and result curves for the read-out align with the experimental read-out data, indicating the correctness of the presented model (detailed analysis in Supplementary Figures S7 and S9).

## 4. Discussion

Recent reviews have demonstrated that platelets can adopt a wide range of functionally and qualitatively distinct states, or different phenotypes, e.g., resting, activated, aggregating, inhibited, and exhausted phenotypes [8,12,46]. However, the commonly accepted classifications of platelet phenotypes and quantitative characterizations of transitions between different platelet phenotypes are poorly described. Moreover, existing hemostasis models mostly focus on platelet adhesion, thrombus formation, or individual biochemical signaling cascades [21,22,31], yet no in silico models have been developed to comprehensively describe the diversity of platelet transitions between different phenotypes. A recently introduced systems biology model describes the differentiation of platelets into aggregating and procoagulant subpopulations; however, it primarily addresses intracellular signaling cascades and is limited to only two phenotypes [47]. At the same time, mathematical modeling is used, for example, to study the dynamics of reversible transitions between phenotypic states of tumor cells with varying resistance to cytotoxic stress [48], and it is also widely applied to assessing the dynamics of growth, death, and differentiation of various T-cell phenotypes [49]. In several studies, it has been shown that dose–response relationships (Hill equations) can be effectively applied to describe various complex signaling cascades [38,39,40,41,50]. For example, this approach has been applied in the analysis of the mitogen-activated protein kinase (MAPK) cascade [51] and in modeling the cell cycle [52]. Accordingly, by using this approach, we built a kinetic model that could be applied for the characterization of platelet transitions between different phenotypes. We provided qualitative characterizations of these transitions as a function of exposure time (rate constants) and the concentrations of active substances (half-maximal effective concentration and Hill coefficient) and demonstrated that the experimental data align with theoretical predictions from the obtained parameters.

The standardization of experiments is extremely important for model development; therefore, first, we established the experimental conditions under which platelet transformations were assessed. The following conditions were established for our experiments: (1) platelet concentration in the buffer should be in the range of 18,000–20,000/mL; (2) [Ca^2+^]_i_ concentration 2 mM; (3) pH of 7.4. All subsequent experiments were performed according to the established conditions. We also showed that platelet aggregation under these conditions directly correlates with the activation of αIIbβ3 integrins, as indicated by PAC-1 binding, which indicates that the parameters of platelet aggregation obtained by the laser diffraction technique could be used for the evaluation of the rapid activation and deactivation kinetics of αIIbβ3 integrins.

For the quantitative characterization of platelet desensitization processes (their transition to the exhausted phenotype [11]), we developed two novel methods to measure and establish key parameters. First, to evaluate the desensitization of platelets from the shape change stage, we applied a low dose of ADP to induce weak activation, followed by a high dose of ADP to induce aggregation after a certain period. Next, platelets were stimulated with a high dose of ADP that activated αIIbβ3 integrins. Then, in the stage of the shape change reaction, the stirrer was turned off, and after a fixed time, it was turned on again. This approach allowed us to estimate whether the already activated integrins, in the absence of platelet interaction, could return to an inactive form (exhausted platelet phenotype). In both cases, the effect was dependent on the duration of reaction interruption and the dose of the agonist. These approaches allowed us to quantitatively characterize the constants of the platelet transition to the exhausted phenotype. Hence, we here establish a mathematical model relying on carefully experimentally determined rate constants, which nevertheless accurately describes all read-out data we could experimentally determine.

cAMP and cGMP, by the activation of the corresponding protein kinases (PKA/PKG), are known as some of the key mediators of platelet inhibitory pathways [45,53]. It is also known that these pathways can induce platelet disaggregation and integrin inactivation when platelets are activated by different agonists [54,55]. However, the quantitative characterization of the processes of platelet inactivation induced by cyclic nucleotides has not been defined. Two questions are important for the characterization of platelet inactivation processes. The first question is which minimum time of receptor-mediated PKA activation (iloprost) is sufficient to induce inactivation processes; the second is to establish the time at which already activated platelets can be inactivated by cyclic nucleotide pathways. Even 5 s of iloprost administration is sufficient for the complete inhibition of ADP-induced platelet aggregation; however, at this time point, the shape change reaction and [Ca^2+^]_i_ concentration are reduced, but not fully inhibited. When iloprost is added, even after 5 s of platelet stimulation by ADP, platelet activation is not fully inhibited, and after 180 s, aggregation (integrin activation) becomes irreversible. It was also demonstrated that the inhibitory effect of iloprost persists for up to 2 min after platelet activation by TRAP, whereas in the case of CRP-XL, this time window is reduced to ~30 s [55]. Interestingly, it has previously been shown that cAMP/PKA activation induces cytoskeletal remodeling even after 10 min following activation [29]. The presented data are in good agreement with proteomic and phosphoproteomic results which have shown that the platelet response to ADP and iloprost is mediated by numerous intracellular signaling pathways, which could be regulated in a time- and concentration-dependent manner [56,57,58]. The presented quantitative data of platelet inhibition (reversible/irreversible) will be important for developing models of intracellular signaling mechanisms responsible for PKA/PKG-mediated processes.

### Limitations of This Study

All in vitro experiments definitely have certain limitations. The proposed model of platelet hemostasis is deliberately simplified and does not include the coagulation cascade, procoagulant or apoptotic platelet phenotypes, secretory phenotypes, micro-particle generation, and clot formation. The laser diffraction method is not feasible for the estimation of rate constants of processes that return cells to the resting phenotype because the light scattering signal for exhausted and inhibitory platelets overlaps with that of resting platelets. Furthermore, we were unable to isolate the disaggregation signal from the signals of other concurrently occurring processes. Therefore, the constants $k_{-1}$, $k_{-4}$, and $k_{7}$ (see Figure 1) were not directly determined experimentally but were instead optimized to best fit the model results to the experimental curves. We also did not directly assess the quantitative effect of cAMP/cGMP activation on the formation of exhausted platelets experimentally. The platelet concentration in our experiments was much less than that in the blood. The developed model also does not account for spatial aspects such as thrombus formation under flow conditions or multiscale factors such as transitions from the cellular to the vascular level. Therefore, our model cannot be directly transformed to whole-blood conditions.

## 5. Conclusions

It is now increasingly recognized that platelets, even under normal physiological conditions and especially in many pathological situations, are not a homogeneous population but represent different phenotypes that are interconnected with reversible and/or irreversible (full aggregation) processes. However, there are no clear definitions of different, especially circulating, platelet phenotypes, and there is only limited information concerning quantitative characteristics of transitions between different types of platelets. In our study, we present a mathematical model that describes the kinetics of platelet transitions between distinct subpopulations in response to ADP and TRAP and calculate the rate constants of these processes. The presented data should help to understand the mechanisms of platelet transition between different phenotypes. Importantly, the theoretically estimated constants of platelet transitions between different phenotypes aligned well with the experimental read-out data, indicating the accuracy of our mathematical model. Nevertheless, even in its current form, the model effectively characterizes key physiological phenotypes within the platelet aggregation cascade and can serve as a modular framework for integration into more comprehensive models. Our findings and the proposed model can also be complemented by data from patients with various pathological conditions, including thrombosis, autoimmune diseases, and inflammation, which would enhance the clinical relevance of the model.

## Figures and Tables

**Table 1:** Dose–response parameters for the shape change response ($V_{sh}$) induced by various agonists.

	ADP	TRAP
${EC}_{50}$, nM	46.8 ± 7.3	204.0 ± 18.7
h (Hill)	1.27 ± 0.08	1.24 ± 0.10
*n*	8	6

**Table 2:** Dose–response parameters for dependence of the initial aggregation rate (integrin αIIbβ3 activation) on agonist concentration.

	ADP	TRAP
${EC}_{50}$, nM	589.8 ± 9.3	2309 ± 233
h (Hill)	3.92 ± 0.61	6.68 ± 0.49
*n*	10	6

**Table 3:** The values of the desensitization rate constant ($k_{5}$) from the shape change state at varying concentrations of the agonist (ADP).

ADP, nM	1000	2000	5000
$k_{5}$, 1/s	0.0019	0.0013	0.0005
τ_1/2_, s	364	554	1280
*n*	5	5	5

**Table 4:** The values of the desensitization rate constant ($k_{6}$) from the shape change state at varying concentrations of the agonist (ADP).

ADP, nM	1000	2000	5000
$k_{6}$, 1/s	0.0279	0.0092	0.0026
τ_1/2_, s	25	76	270
*n*	5	5	5

**Table 5:** Inhibition rate constants for shape change, aggregation, and intracellular calcium dynamics.

	Shape Change	Aggregation	[Ca^2+^]_i_
k, 1/s	0.31 ± 0.04	0.31 ± 0.10	0.085 ± 0.041
τ_1/2_, s	31.28	2.51	18.20
n	7	4	5

**Table 6:** Dose-dependent parameters of iloprost for shape change and aggregation.

	Shape Change ($V_{sh}$)	Aggregation ($V_{agg}$)	Aggregation (${AUC}_{agg}$)
${IC}_{50}$, nM	0.048 ± 0.023	0.21 ± 0.08	0.20 ± 0.07
h	1.52 ± 0.70	2.14 ± 0.91	2.16 ± 0.88
*n*	6	7	7

## Data Availability

The mathematical model is available in our GitHub repository: https://github.com/ldelt/PLT-Phenotypes-Model (accessed on 6 May 2025). The experimental data underlying this article will be shared at reasonable request to the corresponding author.

## Supplementary Materials

The following supporting information can be downloaded at https://www.mdpi.com/article/10.3390/cells14090677/s1, Figure S1: Graphical representation of the analysis of (A) aggregation, (B) shape change, and (C) intracellular calcium dynamics; Figure S2: Effect of pH on aggregation; Figure S3: Fibrinogen concentration is not a limiting factor for aggregation under our experimental conditions; Figure S4: Experimental time-dependent inhibition of shape change by iloprost and SNP; Figure S5: Inhibition of platelet activation by SNP; Figure S6: In the absence of the inhibition constant ($k_{i}$), the suppression of (A) aggregation AUC and (B) aggregation speed is too weak, while (C) the shape change reaction is excessively inhibited; Figure S7: The model using a non-competitive mechanism yields significantly better results than the competitive model; Figure S8: The shape change reaction is not completely inhibited at high iloprost concentrations; Figure S9: Theoretical disaggregation (red) is close to the experimentally observed disaggregation (blue); Table S1: Inhibition rate constants for shape change, aggregation, and intracellular calcium dynamics (SNP); Table S2: Summary table of simulation parameters; Equations (S1)–(S57).
