# Supplementary material for: Experimental and Mathematical Model of Platelet Hemostasis Kinetics
Source: Cells. 2025 May 7;14(9):677. doi: 10.3390/cells14090677 (PMC12071887; doi:10.3390/cells14090677)
Supplement: Supplementary file 1 [file cells-14-00677-s001.zip › cells-3620409-supplementary.pdf]

Supplementary Materials

S1. Analysis of Aggregation, Shape Change, and Calcium Dynamics Using the Laser Diffraction Method

A detailed explanation of the methodological principles is presented in our previous studies [1–3]. Based on the light scattering indicatrix, angles of 1° and 12° were selected for the analysis of aggregation/disaggregation and shape change, respectively. Calcium dynamics were assessed by measuring fluorescence intensity in the FL(527) channel. Aggregation was evaluated using the instantaneous rate at 20 seconds after agonist addition (V_{agg}) (Figure S1A). Disaggregation was quantified as the percentage reduction in signal intensity following the maximum response ($Disagg$). The area under the curve (AUC_{agg}) was used as an integral measure of both aggregation and disaggregation over the first 120 seconds of the process. The shape change response was assessed based on the instantaneous rate at 5 seconds (V_{sh}) (Figure S1B). Intracellular calcium mobilization dynamics were evaluated using the area under the fluorescence curve over the first 20 seconds (Figure S1C).

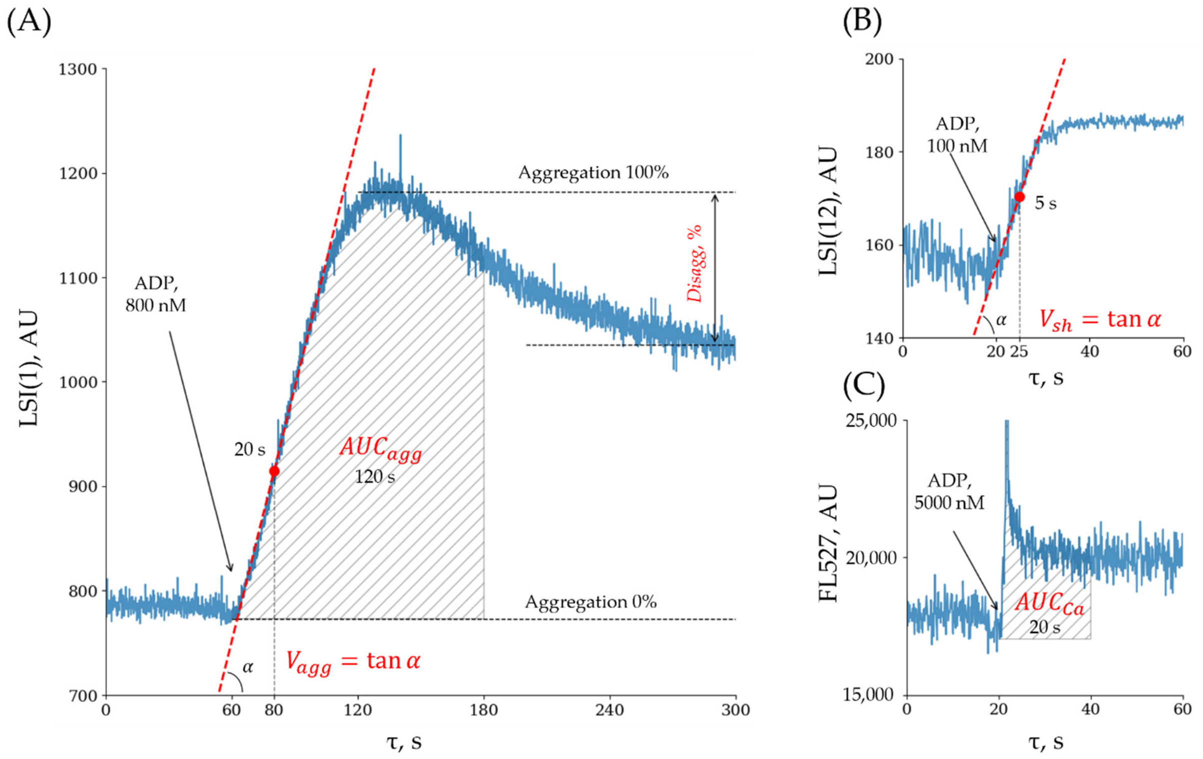

Figure S1. Graphical representation of the analysis of (A) aggregation, (B) shape change, and (C) intracellular calcium dynamics. PRP was added to the cuvette containing 1000 μL of HEPES buffer (2 mM Ca^{2+}) with continuous stirring (1200 rpm) at 37°C (final platelet concentration: 2×10^7 cells/mL). Reactions were induced by ADP or TRAP. (A) V_{agg}, AUC_{agg}, (B) V_{sh}, and (C) AUC_{Ca} were analyzed using the original software LaSca_32v.1750. (A) Disaggregation ($Disagg$) was assessed as the percentage decrease in LSI from its maximum.

S2. The Mathematical Approach Used to Calculate the Instantaneous Rate Constants in the Final Version of the Model

Below is the mathematical description of the model that yielded the closest results to the experimental data. For model comparisons, see Figure 10 of the article. A complete list of equations is available in Supplementary Section 8. ADP is used as the agonist and iloprost as the antagonist, as they have the most experimentally determined parameters (see Supplementary Table S2).

Calculation of the instantaneous rate constants for shape change (k_1) and integrin activation (k_2)

The rate constants for shape change (k_1) and integrin αIIbβ$_3$ activation (k_2) were determined by evaluating the inhibition constant k_i (Equation 1) and applying a modified Hill equation (Equation 2) that incorporates inhibition via a non-competitive mechanism. Equations (S1-S2):

$$k_i = IC_{(50\_Ilo)}, \quad (S1)$$

$$k = k_{max} \frac{[ADP]^{h_{ADP}} / \left(1 + \frac{[Ilo]}{k_i}\right)}{[ADP]^{h_{ADP}} + EC_{(50\_ADP)}^{h_{ADP}}}, \quad (S2)$$

where k_i is the inhibition constant (nM), $IC_{(50\_Ilo)}$ is the half-maximal inhibitory concentration of iloprost (nM), $[ADP]$ is the ADP concentration (nM), $[Ilo]$ is the iloprost concentration (nM), $EC_{(50\_ADP)}$ is the half-maximal effective concentration of ADP (nM), and h_{ADP} is the Hill coefficient for ADP.

Calculation of the instantaneous rate constants for desensitization (k_5, k_6) and disaggregation (k_7)

The baseline reaction rates in the absence of aggregation antagonists were determined using the following Equation (S3):

$$k_0 = k_{max} \frac{IC_{(50\_ADP)}^{h_{ADP}}}{[ADP]^{h_{ADP}} + IC_{(50\_ilo)}^{h_{ADP}}}, \quad (S3)$$

where k_0 is the rate constant for desensitization/disaggregation in the absence of aggregation antagonists (nM); k_{max} is the experimentally determined maximum value of the rate constant k_0 (1/s); $IC_{(50\_ADP)}$ is the half-maximal inhibitory concentration of ADP (nM); [ADP] is the ADP concentration (nM); and h_{ADP} is the Hill coefficient for ADP.

Next, the inhibition constant (k_{ia}) was determined as in Equation (S1), and the antagonist-induced desensitization/disaggregation rate (k_a) was calculated as in Equation (S2). Equations (S4-S5):

$$k_{ia} = IC_{(50\_ADP)}, \quad (S4)$$

$$k_a = k_{max} \frac{[Ilo]^{h_{Ilo}} / \left(1 + \frac{[ADP]}{k_{ia}}\right)}{[Ilo]^{h_{Ilo}} + EC_{(50\_ilo)}^{h_{Ilo}}}, \quad (S5)$$

where k_{ia} is the inhibition constant for k_a (nM); k_a is the antagonist-induced disaggregation rate constant (1/s); $IC_{(50\_ADP)}$ is the half-maximal inhibitory concentration of ADP (nM); $EC_{(50_{Ilo})}$ is the half-maximal effective concentration of Ilo (nM); and h_{Ilo} is the Hill coefficient for Ilo.

Finally, the values of the constants k_5, k_6, and k_7 were determined as the sum of the corresponding values of k_0 and k_a. Equation (S6):

$$k = k_0 + k_a \quad (S6)$$

Determination of the instantaneous rate constants for aggregation (k_3), inhibition of resting platelets (k_4), and the return of platelets from the inhibitory to the resting state (k_{-4})

The basic form of the Hill equation was used: Equation (S7) for k_3, k_{-4} and Equation (S8) for k_4. Equations (S7-S8):

$$k = k_{max}\frac{[ADP]^{h_{ADP}}}{[ADP]^{h_{ADP}} + EC_{(50\_ADP)}^{h_{ADP}}}, \tag{S7}$$

$$k = k_{max}\frac{[Ilo]^{h_{Ilo}}}{[Ilo]^{h_{Ilo}} + EC_{(50\_Ilo)}^{h_{Ilo}}}, \tag{S8}$$

S3. The Effect of pH on Platelet Aggregation

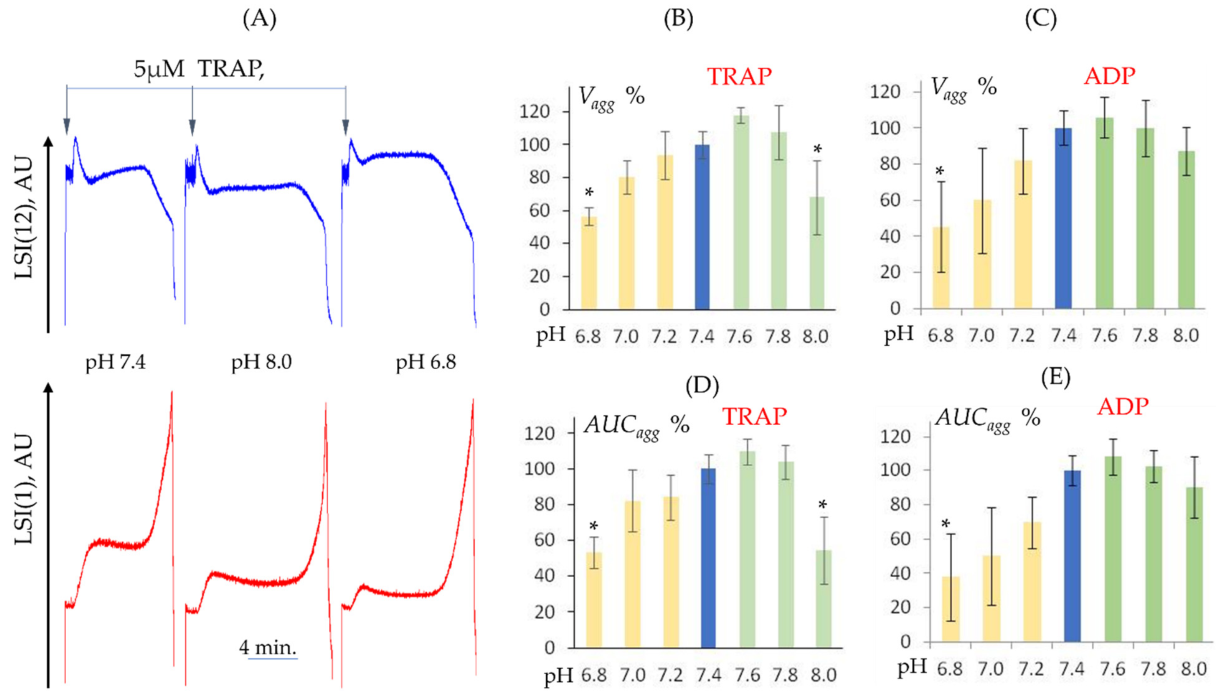

Figure S2. Effect of pH on aggregation. Platelets were activated with ADP (5 μM) or TRAP (5 μM), with responses at pH 7.4 in the presence of 5 μM agonist used as the reference (100%). (A) Experimental traces of shape change (top) and aggregation (bottom). Shape change remained unaffected across the tested pH range. Statistically significant differences in (B, C) V_{agg} and (D, E) AUC_{agg} were detected only at pH values outside the physiological range (7.0–7.8): at pH 6.8 for platelets stimulated with (C, E) ADP and (B, D) TRAP, and at pH 8.0 for platelets activated with (B, D) TRAP. One-way ANOVA, Dunnet's test, *, $p<0.05$.

S4. Fibrinogen Concentration

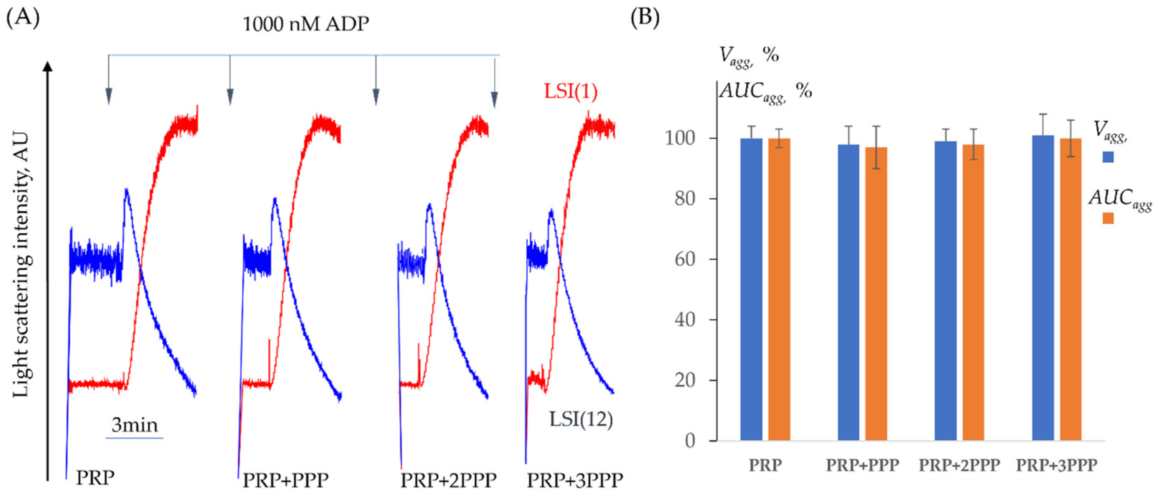

Figure S3. Fibrinogen concentration is not a limiting factor for aggregation under our experimental conditions. (A) The experimental dependencies of aggregation (LSI(1)) and shape change (LSI(12)) are identical in the control condition (PRP: 50 μL PRP + 950 μL HEPES) and when PPP is added to the medium in an equal or greater volume relative to PRP (PRP+PPP: 50 μL PRP + 50 μL PPP + 900 μL HEPES, PRP+2PPP: 50 μL PRP + 100 μL PPP + 850 μL HEPES, PRP+3PPP: 50 μL PRP + 150 μL PPP + 850 μL HEPES). (B) No differences were observed between the aggregation of control and experimental samples. One-way ANOVA, Dunnet's test.

S5. The shape change response is completely inhibited within 5 seconds of exposure to cAMP/cGMP activators

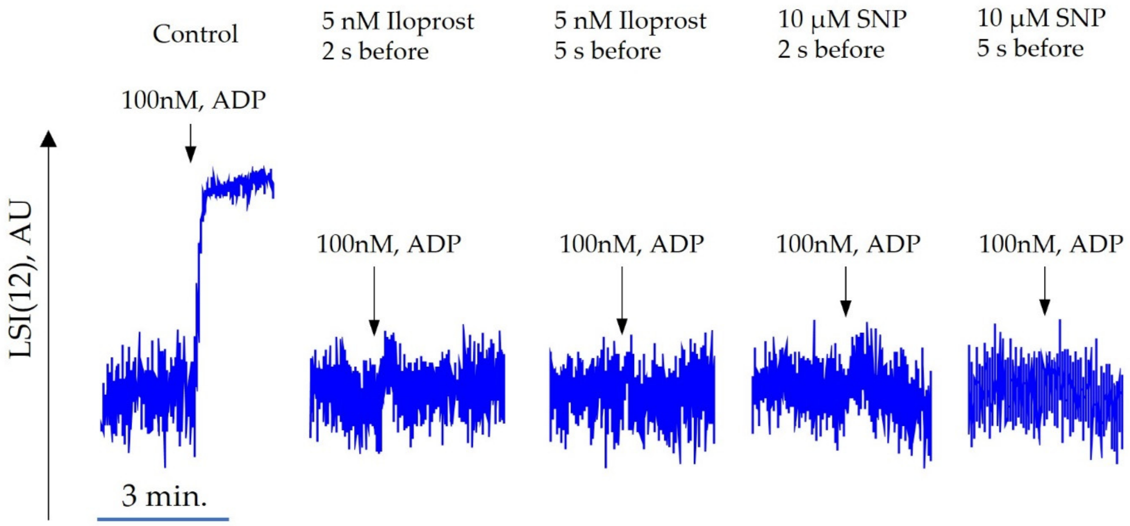

Figure S4. Experimental time-dependent inhibition of shape change by iloprost and SNP. A weak shape change response was observed after 2 seconds of exposure to iloprost or SNP, while complete inhibition occurred within 5-10 seconds.

S6. Inhibition of platelet activation by SNP

The experimental protocol followed the same approach as described for iloprost (see Section 3.3.3 of the article). The SNP concentration used was 10 μM. The shape change response was fully inhibited within the first 5–10 seconds of SNP exposure (Figure S4, Figure S5B). A 5-second exposure to SNP was sufficient to suppress aggregation (Figure S5A upper panel, D). The calcium response declined more gradually, reaching only 35–40% of the control level (Figure S5A bottom panel, C). The addition of SNP after platelet activation led to intracellular calcium efflux (Figure S5C upper right corner). The extent of disaggregation decreased as the time interval between platelet activation and SNP addition increased (Figure S5E). The inhibition rate constants for shape change, aggregation, suppression of $[Ca^{2+}]_i$ dynamics, and disaggregation are presented in Table S1.

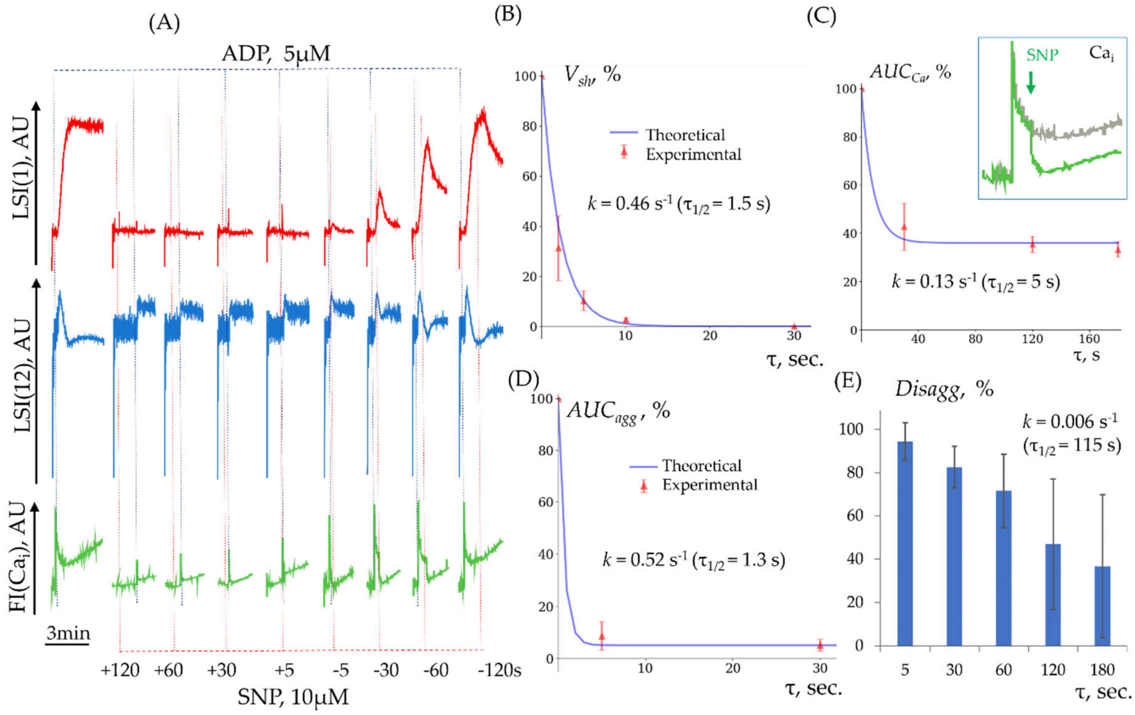

Figure S5. Inhibition of platelet activation by SNP. (A) Temporal profiles of platelet aggregation (LSI(1), red), shape change (LSI(12), blue), and intracellular calcium dynamics (green) are displayed before and after administration of SNP. (B) Rate of shape change reaction (V_{sh}) versus time between SNP addition and ADP-induced activation. In the upper right corner, calcium efflux from the cytoplasm is shown upon addition of SNP after platelets activation (green), compared to the control condition without SNP (grey). (D) Area under aggregation curve (AUC_{agg}) versus time between SNP addition and ADP-induced activation. (E) Disaggregation ($Disagg$) versus time between initial activation and SNP addition. V_{sh}, AUC_{agg}, AUC_{Ca} are normalized to control experiments without inhibitor (100%).

Table S1. Inhibition rate constants for shape change, aggregation, and intracellular calcium dynamics (SNP)

	**Shape Change**	**Aggregation**	**$[Ca^{2+}]_i$**	**Disaggregation**
k, 1/s	0.46 ± 0.09	0.52 ± 0.14	0.13 ± 0.01	0.006
$\tau_{1/2}$, s	1.55	1.51	12.12	115
n	3	4	3	6

S7. Determination of the model parameters

All parameters used in the model are presented in Table S2.

Table S2. Summary table of simulation parameters

Parameter	Mean	Parameter	Mean	n	Method for finding
$k_{(\max\_1)}$	0.19 s^{-1}			41	Direct experiment
$EC_{(50\_ADP\_1)}$	46.8 nM	$h_{(ADP\_1)}$	1.27	8	Direct experiment [1]
$IC_{(50\_Ilo\_1)}$	0.048 nM	$h_{(Ilo\_1)}$	1.52	6	Direct experiment [1]
$k_{(\max\_2)}$	0.016 s^{-1}			14	Direct experiment [1]
$EC_{(50\_ADP\_2)}$	589.8 nM	$h_{(ADP\_2)}$	3.92	10	Direct experiment [1]
$IC_{(50\_Ilo\_2)}$	0.21 nM	$h_{(Ilo\_2)}$	2.14	7	Direct experiment [1]
$k_{(\max\_3)}$	0.032 s^{-1}		-		Rate is limited [2]
$EC_{(50\_ADP\_3)}$	589.8 nM	$h_{(ADP\_3)}$	3.92		Rate is limited [2]
$k_{(\max\_5)}$	0.0028 s^{-1}			5	Direct experiment [1]
$IC_{(50\_ADP\_5)}$	1840 nM	$h_{(ADP\_5)}$	1.4	5	Direct experiment [1]
$EC_{(50\_Ilo\_5)}$	0.048 nM	$h_{(Ilo\_5)}$	1.52	6	Indirect experiment [3]
$k_{(\max\_6)}$	0.055 s^{-1}			5	Direct experiment [1]
$IC_{(50\_ADP\_6)}$	990 nM	$h_{(ADP\_6)}$	1.9	5	Direct experiment [1]
$EC_{(50\_Ilo\_6)}$	0.21 nM	$h_{(Ilo\_6)}$	2.14	7	Indirect experiment [3]
$k_{(\max\_7)}$	0.024 s^{-1}				Optimized [4]
$IC_{(50\_ADP\_7)}$	974.68 nM	$h_{(ADP\_7)}$	1.00		Optimized [4]
$EC_{(50\_Ilo\_7)}$	0.21 nM	$h_{(Ilo\_7)}$	2.14	7	Indirect experiment [3]
$k_{(\max\_4)}$	0.031 s^{-1}				Optimized [4]
$EC_{(50\_Ilo\_4)}$	0.14 nM	$h_{(Ilo\_12)}$	1.52		Optimized [4]
$k_{(\max\_-4)}$	0.036 s^{-1}				Optimized [4]
$EC_{(50\_ADP\_-4)}$	14864.19 nM	$h_{(ADP\_21)}$	1.00		Optimized [4]
$k_{(\max\_-1)}$	0.0015 s^{-1}				Optimized [4]

[1] Determined experimentally.

[2] Since αIIbβ3 integrin activation is the rate-limiting step under the specified experimental conditions (see Sections 3.2.1 and 3.3.1), $k_{(\max\_3)}$ is set to twice the value of $k_{(\max\_2)}$, with $EC_{(50\_ADP\_3)}$ and $h_{(ADP\_3)}$ for k_3 assigned the same values as those for k_2.

[3] The values of $EC_{(50\_Ilo)}$ and h_{Ilo} for k_5 and k_6 reverse reactions are set equal to the corresponding $IC_{(50\_Ilo)}$ и h_{Ilo} values for the respective forward reactions. For k_7, the same parameters as for k_6 were assigned.

[4] The parameters were calculated through optimization using basin-hopping (BH) and L-BFGS methods. For k_4, the search boundaries were set based on the inhibition parameters of shape change and aggregation.

The laser analyzer signal is proportional to the square root of cell concentration

The increase in particle size within the suspension enhances near-angle light scattering signals, which is what we observe during aggregation measurements. However, this effect is counterbalanced by the concurrent decrease in particle concentration, which reduces the overall signal intensity. This dual dependence varies with detection angle and has been addressed in detail in Supplementary Materials of our previous work, where we outlined the physical principles of the measurement technique [2]. Since it is known that at the LSI(1) angle, the concentration dependence of light scattering is well approximated by a square root function, we applied a square root transformation ($\sqrt{N_{agg}}$) to correlate the simulated percentage of cells in the aggregating phenotype (N_{agg}) with experimental aggregation signal.

Resting to sphered and thrombotic platelets

All parameters were determined experimentally: $k_{(\max\_1)}$, $EC_{(50\_ADP\_1)}$, $h_{(ADP\_1)}$, $IC_{(50\_Ilo\_1)}$, $h_{(Ilo\_1)}$ and $k_{(\max\_2)}$, $EC_{(50\_ADP\_2)}$, $h_{(ADP\_3)}$, $IC_{(50\_Ilo\_2)}$, $h_{(Ilo\_2)}$ (See Sections 3.3.1 and 3.3.3 of the article).

Resting to inhibitory platelets and reverse

Currently, we are unable to experimentally quantify the reverse transition of platelets from the inhibitory back to the resting state for two main reasons: the overlapping signals in laser analyzer measurements and the technical impossibility of removing the inhibitory stimulus from the medium. As an initial approximation, we used the maximal reaction rate $k_{(\max\_4)}$ and dose-dependence parameters ($EC_{(50\_Ilo\_4)}$ and $h_{(Ilo\_4)}$) based on experimentally determined values of k, EC_{50_Ilo} and h_{Ilo} obtained from aggregation and shape change inhibition experiments (See Table 6 of the article). The modeled inhibition of aggregation and shape change deviated significantly from the experimental observations (Figure S6). Although the rate of aggregation inhibition was substantially higher than the experimental measurements, the response was not fully suppressed, contradicting experimental findings (see Figure 8A,D and Figure 9B,D in the main text). The inhibition of shape change was overly pronounced.

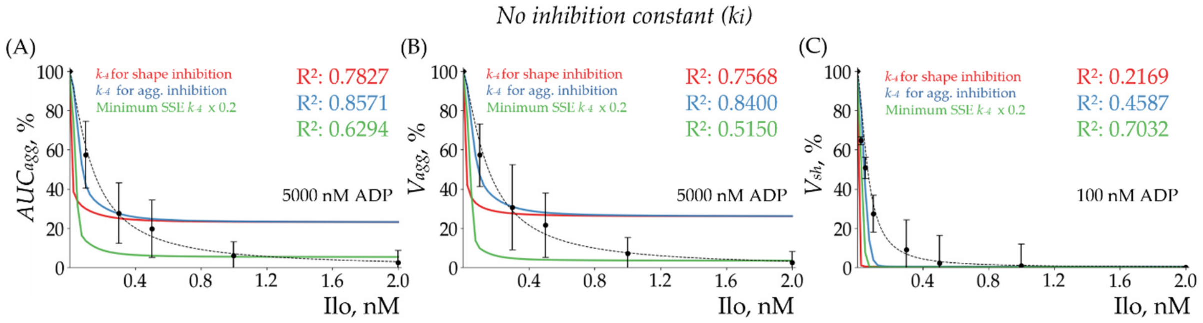

Figure S6. In the absence of the inhibition constant (k_i), the suppression of (A) aggregation AUC and (B) aggregation speed is too weak, while (C) the shape change reaction is excessively inhibited. The reverse reaction parameters (return of inhibited platelets to resting state) were optimized using basin-hopping (BH) and L-BFGS methods. R^2 is the coefficient of determination. When using rate constants k_4 for shape change inhibition (red) and aggregation suppression (blue), the minimal SSE and maximal coefficient of determination (R^2) values were achieved with partial platelet inhibition. Complete inhibition required at least a 5-fold reduction of the optimal k_{-4} value (green).

Considering that iloprost may affect already activated platelets, we introduced an inhibition constant (k_i) into the model to reduce the rate of direct reactions through either competitive or non-competitive mechanisms. It was crucial to identify the mathematical formalism describing how activators of cyclic nucleotide systems suppress platelet activation reactions [4]. The intracellular molecular mechanisms

responsible for the transition between different platelet phenotypes are beyond the scope of this study. We focus on a mathematical approach that provides a simplified representation of the kinetics underlying platelet transitions between phenotypes. Thus, although cyclic nucleotides are not considered competitive inhibitors from a biochemical standpoint, the intracellular signaling network is highly intricate, and therefore, this approach cannot be dismissed without further verification. Our analysis revealed that the suppression of ADP-induced activation is most accurately described by a non-competitive inhibition model (Figure S7).

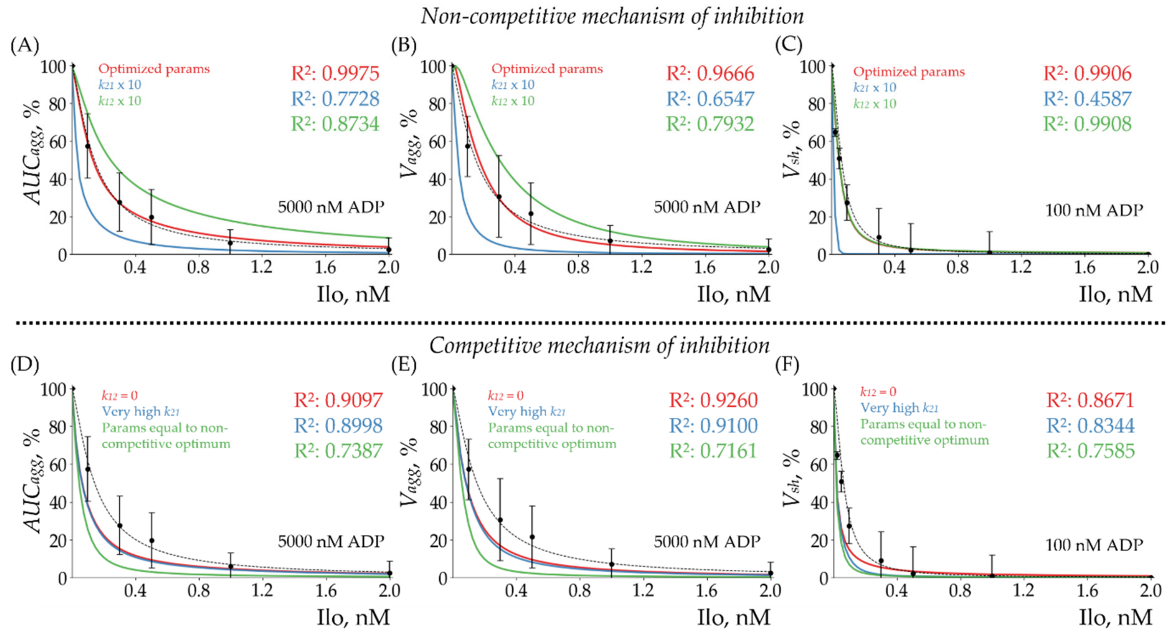

Figure S7. The model using a non-competitive mechanism yields significantly better results than the competitive model. For the non-competitive model, deviations from the optimal balance between rate constants k_4 and k_{-4} substantially worsen the results for (A) AUC_{agg}, (B) V_{agg}, and (C) V_{sh}. In the competitive model, the inhibitory phenotype is excessive and leads to overly strong inhibition (green). Removing inhibitory platelets by setting k_4 to zero (red) or using very high k_{-4} values (blue) improves the results, but even under these conditions (D) AUC_{agg}, (E) V_{agg}, and (F) V_{sh} remain excessively suppressed. For the non-competitive model, the reaction parameters k_4 and k_{-4} were optimized using basin-hopping and L-BFGS methods. For the competitive model, the attempt to optimize parameters while maintaining the inhibitory phenotype is described in the main text (see Figure 10). R^2 is the coefficient of determination.

While this approach successfully reproduces the incomplete suppression of shape change by iloprost at high ADP concentrations (Figure S8), a notable divergence remains in the response kinetics. Experimentally, platelets exhibit an abrupt transition to the shape change state, whereas modeled cells show a gradual response. The best kinetic match is achieved by the model excluding the inhibition constant k_i, though this version fails to replicate experimental behavior at low agonist concentrations (see Figure S6C).

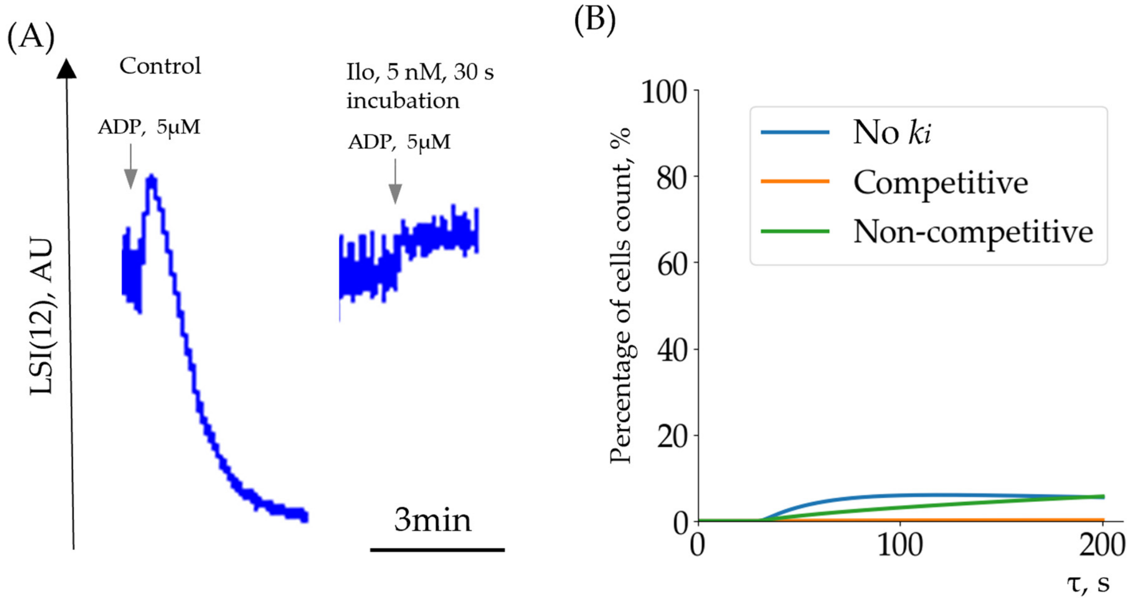

Figure S8. The shape change reaction is not completely inhibited at high iloprost concentrations. (A) After 30-second incubation of platelets with high iloprost concentration, a high ADP dose still produces shape change detectable by LSI(12). (B) The non-competitive model also shows incomplete inhibition of the shape change reaction.

The introduction of the inhibition constant (k_i) does not allow us to distinguish between the formation of inhibitory platelets and the suppression of the reaction rates k_1 and k_2. Therefore, the parameters $k_{(\max\_4)}$, $EC_{(50\_ADP\_4)}$, $h_{(ADP\_4)}$, $k_{(-4)}$, $EC_{(50\_ADP\_-4)}$ and $h_{(ADP\_-4)}$ were optimized using basin-hopping (BH) for global optimization and L-BFGS for local optimization. Since the rates of disaggregation and exhausted platelet formation occur on a significantly slower timescale than the initial activation reactions, we were able to optimize the parameters for these processes independently.

Shape change, thrombotic and aggregating platelets to exhausted and resting platelets

The maximum rate constants and dose-dependence parameters for transitions to the exhausted phenotype from both shape change and thrombotic states were determined experimentally: $k_{(\max\_5)}$, $IC_{(50\_ADP\_5)}$, $h_{(ADP\_5)}$, $k_{(\max\_6)}$, $IC_{(50\_ADP\_6)}$, $h_{(ADP\_6)}$ (see Section 3.3.2). However, accurately estimating the disaggregation parameters (k_7), proved challenging in our experimental conditions, as this slow process is coupled with multiple parallel reactions—particularly the reactivation of previously exhausted/disaggregated platelets (k_{-1}). To avoid overparameterization of the model at this stage, we set k_{-1} equal to $k_{(\max\_-1)}$ and determined it simultaneously with k_{7_max}, $IC_{(50\_ADP\_7)}$ and $h_{(ADP\_7)}$ using BH and L-BFGS optimization (Figure S9). This approach was implemented under inhibitor-free conditions, thereby eliminating the need to concurrently estimate iloprost dose-dependence parameters.

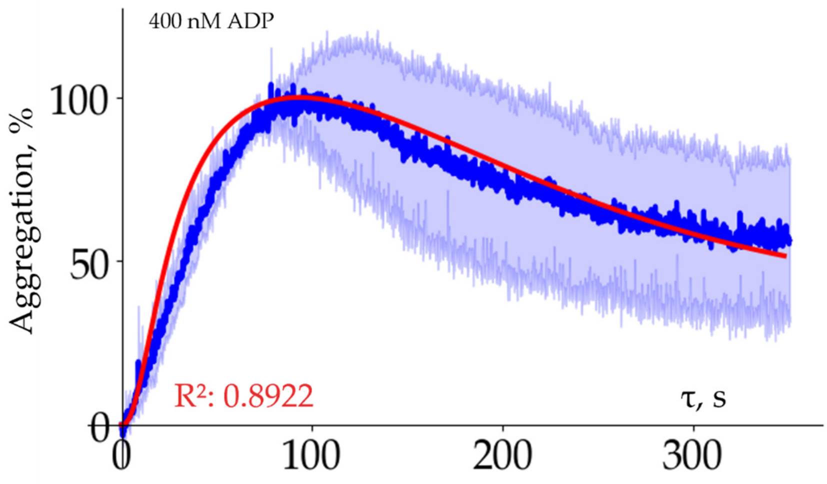

Figure S9. Theoretical disaggregation (red) is close to the experimentally observed disaggregation (blue).

The analysis of iloprost-dependent desensitization presents significant challenges. These dependencies cannot be adequately described by a simple Hill equation incorporating both inhibitor and agonist effects, as applying Equation S2 in its basic form predicts complete absence of disaggregation in the absence of iloprost—a finding inconsistent with experimental observations. Furthermore, under our experimental conditions, it remains technically difficult to independently assess the relative contributions of both factors to the disaggregation process. For modeling iloprost-dependent platelet desensitization, we adopted the following dose-response parameters: ($EC_{(50\_Ilo\_5)}$, $h_{(Ilo\_5)}$, $EC_{(50\_Ilo\_6)}$, and $h_{(Ilo\_6)}$) were set equal to previously determined $IC_{(50\_Ilo\_1)}$, $h_{(Ilo\_1)}$, $IC_{(50\_Ilo\_2)}$, and $h_{(Ilo\_2)}$ correspondingly. $EC_{(50\_Ilo\_7)}$ and $h_{(Ilo\_7)}$ were assumed identical to $EC_{(50\_Ilo\_6)}$ and $h_{(Ilo\_6)}$. Additionally, we accounted for baseline disaggregation by incorporating additive effects (Equations S4-S6). While this approach represents a simplification, it provides a reasonable first approximation of platelet exhaustion and disaggregation dynamics.

S8. List of all equations of the model

Below is the complete list of equations used in our model to determine the instantaneous reaction rate constants.

- Non-competitive, Equations (S9-S28):

$$k_{(i\_1)} = IC_{(50\_Ilo\_1)}\,, \quad (S9)$$

$$k_1 = k_{(\max\_1)} \frac{[ADP]^{h_{(ADP\_1)}} / \left(1 + \frac{[Ilo]}{k_{(i\_1)}}\right)}{[ADP]^{h_{(ADP\_1)}} + EC_{(50\_ADP\_1)}^{h_{(ADP\_1)}}}, \quad (S10)$$

$$k_{(i\_2)} = IC_{(50\_Ilo\_2)}\,, \quad (S11)$$

$$k_2 = k_{(\max\_2)} \frac{[ADP]^{h_{(ADP\_2)}} / \left(1 + \frac{[Ilo]}{k_{(i\_2)}}\right)}{[ADP]^{h_{(ADP\_2)}} + EC_{(50\_ADP\_2)}^{h_{(ADP\_2)}}}, \quad (S12)$$

$$k_3 = k_{(\max\_3)} \frac{[ADP]^{h_{(ADP\_3)}}}{[ADP]^{h_{(ADP\_3)}} + EC_{(50\_ADP\_3)}^{h_{(ADP\_3)}}}, \quad (S13)$$

$$k_4 = k_{(\max\_4)} \frac{[Ilo]^{h_{(Ilo\_4)}}}{[Ilo]^{h_{(Ilo\_4)}} + EC_{(50\_Ilo\_4)}^{h_{(Ilo\_4)}}}, \tag{S14}$$

$$k_{-4} = k_{(\max\_-4)} \frac{[ADP]^{h_{(ADP\_-4)}}}{[ADP]^{h_{(ADP\_-4)}} + EC_{(50\_ADP\_-4)}^{h_{(ADP\_-4)}}}, \tag{S15}$$

$$k_{(0\_5)} = k_{(\max\_5)} \frac{IC_{(50\_ADP\_5)}^{h_{(ADP\_5)}}}{[ADP]^{h_{(ADP\_5)}} + IC_{(50\_ADP\_5)}^{h_{(ADP\_5)}}}, \tag{S16}$$

$$k_{(i\_a\_5)} = IC_{(50\_ADP\_5)}, \tag{S17}$$

$$k_{(a\_5)} = k_{(\max\_5)} \frac{[Ilo]^{h_{(Ilo\_5)}} \Big/ \left(1 + \frac{[ADP]}{k_{(i\_a\_5)}}\right)}{[Ilo]^{h_{(Ilo\_5)}} + EC_{(50\_Ilo\_5)}^{h_{(Ilo\_5)}}}, \tag{S18}$$

$$k_5 = k_{(0\_5)} + k_{(a\_5)}, \tag{S19}$$

$$k_{(0\_6)} = k_{(\max\_6)} \frac{IC_{(50\_ADP\_6)}^{h_{(ADP\_6)}}}{[ADP]^{h_{(ADP\_6)}} + IC_{(50\_ADP\_6)}^{h_{(ADP\_6)}}}, \tag{S20}$$

$$k_{(i\_a\_6)} = IC_{(50\_ADP\_6)}, \tag{S21}$$

$$k_{(a\_6)} = k_{(\max\ \_6)} \frac{[Ilo]^{h_{(Ilo\_6)}} \Big/ \left(1 + \frac{[ADP]}{k_{(i\_a\_6)}}\right)}{[Ilo]^{h_{(Ilo\_6)}} + EC_{(50\_Ilo\_6)}^{h_{(Ilo\_6)}}}, \tag{S22}$$

$$k_6 = k_{(0\_6)} + k_{(a\_6)}, \tag{S23}$$

$$k_{(0\_7)} = k_{(\max\_7)} \frac{IC_{(50\_ADP\_7)}^{h_{(ADP\_7)}}}{[ADP]^{h_{(ADP\_7)}} + IC_{(50\_ADP\_7)}^{h_{(ADP\_7)}}}, \tag{S24}$$

$$k_{(i\_a\_7)} = IC_{(50\_ADP\_7)}, \tag{S25}$$

$$k_{(a\_7)} = k_{(\max\ \_7)} \frac{[Ilo]^{h_{(Ilo\_7)}} \Big/ \left(1 + \frac{[ADP]}{k_{(i\_a\_7)}}\right)}{[Ilo]^{h_{(Ilo\_7)}} + EC_{(50\_Ilo\_7)}^{h_{(Ilo\_7)}}}, \tag{S26}$$

$$k_7 = k_{(0\_7)} + k_{(a\_7)}, \tag{S27}$$

$$k_{-1} = k_{(max\_-1)} \tag{S28}$$

- Competitive, Equations (S29-S48):

$$k_{(i\_1)} = \frac{IC_{(50\_Ilo\_1)}}{\left(\frac{[ADP]}{EC_{(50\_ADP\_1)}}\right)^{h_{(Ilo\_1)}} + 1}, \tag{S29}$$

$$k_1 = k_{(\max\_1)} \frac{[ADP]^{h_{(ADP\_1)}}}{[ADP]^{h_{(ADP_1)}} + EC_{(50\_ADP\_1)}^{h_{(ADP\_1)}} \left(1 + \frac{[Ilo]}{k_{(i\_1)}}\right)}, \tag{S30}$$

$$k_{(i\_2)} = \frac{IC_{(50\_Ilo\_2)}}{\left(\frac{[ADP]}{EC_{(50\_ADP\_2)}}\right)^{h_{(Ilo\_2)}} + 1}, \tag{S31}$$

$$k_2 = k_{(\max\_2)} \frac{[ADP]^{h_{(ADP\_1)}}}{[ADP]^{h_{(ADP\_2)}} + EC_{(50\_ADP\_2)}^{h_{(ADP\_2)}} \left(1 + \frac{[Ilo]}{k_{(i\_2)}}\right)}, \quad (S32)$$

$$k_3 = k_{(\max\_3)} \frac{[ADP]^{h_{(ADP\_3)}}}{[ADP]^{h_{(ADP\_3)}} + EC_{(50\_ADP\_3)}^{h_{(ADP\_3)}}}, \quad (S33)$$

$$k_4 = k_{(\max\_4)} \frac{[Ilo]^{h_{(Ilo\_4)}}}{[Ilo]^{h_{(Ilo\_4)}} + EC_{(50\_Ilo\_4)}^{h_{(Ilo\_4)}}}, \quad (S34)$$

$$k_{-4} = k_{(\max\_-4)} \frac{[ADP]^{h_{(ADP\_-4)}}}{[ADP]^{h_{(ADP\_-4)}} + EC_{(50\_ADP\_-4)}^{h_{(ADP\_-4)}}}, \quad (S35)$$

$$k_{(0\_5)} = k_{(\max\_5)} \frac{IC_{(50\_ADP\_5)}^{h_{(ADP\_5)}}}{[ADP]^{h_{(ADP\_5)}} + IC_{(50\_ADP\_5)}^{h_{(ADP\_5)}}}, \quad (S36)$$

$$k_{(i\_a\_5)} = \frac{IC_{(50\_ADP\_5)}}{\left(\frac{[Ilo]}{EC_{(50\_Ilo\_5)}}\right)^{h_{(ADP\_5)}} + 1}, \quad (S37)$$

$$k_{(a\_5)} = k_{(\max\_5)} \frac{[Ilo]^{h_{(Ilo\_5)}}}{[Ilo]^{h_{(Ilo\_5)}} + EC_{(50\_Ilo\_5)}^{h_{(Ilo\_5)}} \left(1 + \frac{[ADP]}{k_{(i\_a\_5)}}\right)}, \quad (S38)$$

$$k_6 = k_{(0\_6)} + k_{(a\_6)}, \quad (S39)$$

$$k_{(0\_6)} = k_{(\max\_6)} \frac{IC_{(50\_ADP\_6)}^{h_{(ADP\_6)}}}{[ADP]^{h_{(ADP\_6)}} + IC_{(50\_ADP\_6)}^{h_{(ADP\_6)}}}, \quad (S40)$$

$$k_{(i\_a\_6)} = \frac{IC_{(50\_ADP\_6)}}{\left(\frac{[Ilo]}{EC_{(50\_Ilo\_6)}}\right)^{h_{(ADP\_6)}} + 1}, \quad (S41)$$

$$k_{(a\_6)} = k_{(\max\_6)} \frac{[Ilo]^{h_{(Ilo\_6)}}}{[Ilo]^{h_{(Ilo\_6)}} + EC_{(50\_Ilo\_6)}^{h_{(Ilo\_6)}} \left(1 + \frac{[ADP]}{k_{(i\_a\_6)}}\right)}, \quad (S42)$$

$$k_6 = k_{(0\_6)} + k_{(a\_6)}, \quad (S43)$$

$$k_{(0\_7)} = k_{(\max\_7)} \frac{IC_{(50\_ADP\_7)}^{h_{(ADP\_7)}}}{[ADP]^{h_{(ADP\_7)}} + IC_{(50\_ADP\_7)}^{h_{(ADP\_7)}}}, \quad (S44)$$

$$k_{(i\_a\_7)} = \frac{IC_{(50\_ADP\_7)}}{\left(\frac{[Ilo]}{EC_{(50\_Ilo\_7)}}\right)^{h_{(ADP\_7)}} + 1}, \quad (S45)$$

$$k_{(a\_7)} = k_{(\max\_7)} \frac{[Ilo]^{h_{(Ilo\_7)}}}{[Ilo]^{h_{(Ilo\_7)}} + EC_{(50\_Ilo\_7)}^{h_{(Ilo\_7)}} \left(1 + \frac{[ADP]}{k_{(i\_a\_7)}}\right)}, \quad (S46)$$

$$k_7 = k_{(0\_7)} + k_{(a\_7)} \quad (S47)$$

$$k_{-1} = k_{(max\_-1)} \quad (S48)$$

- Without k_i, Equations (S48-S57):

$$k_1 = k_{(\max\_1)} \frac{[ADP]^{h(ADP\_1)}}{[ADP]^{h(ADP\_1)} + EC_{(50\_ADP\_1)}^{h(ADP\_1)}}, \quad (S49)$$

$$k_2 = k_{(\max\_2)} \frac{[ADP]^{h(ADP\_2)}}{[ADP]^{h(ADP\_2)} + EC_{(50\_ADP\_2)}^{h(ADP\_2)}}, \quad (S50)$$

$$k_3 = k_{(\max\_3)} \frac{[ADP]^{h(ADP\_3)}}{[ADP]^{h(ADP\_3)} + EC_{(50\_ADP\_3)}^{h(ADP\_3)}}, \quad (S51)$$

$$k_4 = k_{(\max\_4)} \frac{[Ilo]^{h(Ilo\_4)}}{[Ilo]^{h(Ilo\_4)} + EC_{(50\_Ilo\_4)}^{h(Ilo\_4)}}, \quad (S52)$$

$$k_{-4} = k_{(\max\_-4)} \frac{[ADP]^{h(ADP\_-4)}}{[ADP]^{h(ADP\_-4)} + EC_{(50\_ADP\_-4)}^{h(ADP\_-4)}}, \quad (S53)$$

$$k_5 = k_{(\max\_5)} \frac{IC_{(50\_ADP\_5)}^{h(ADP\_5)}}{[ADP]^{h(ADP\_5)} + IC_{(50\_ADP\_5)}^{h(ADP\_5)}}, \quad (S54)$$

$$k_6 = k_{(\max\_6)} \frac{IC_{(50\_ADP\_6)}^{h(ADP\_6)}}{[ADP]^{h(ADP\_6)} + IC_{(50\_ADP\_6)}^{h(ADP\_6)}}, \quad (S55)$$

$$k_7 = k_{(\max\_7)} \frac{IC_{(50\_ADP\_7)}^{h(ADP\_7)}}{[ADP]^{h(ADP\_7)} + IC_{(50\_ADP\_7)}^{h(ADP\_7)}}, \quad (S56)$$

$$k_{-1} = k_{(max\_-1)} \quad (S57)$$

References

1. Mindukshev, I.; Gambaryan, S.; Kehrer, L.; Schuetz, C.; Kobsar, A.; Rukoyatkina, N.; Nikolaev, V.O.; Krivchenko, A.; Watson, S.P.; Walter, U.; et al. Low Angle Light Scattering Analysis: A Novel Quantitative Method for Functional Characterization of Human and Murine Platelet Receptors. *Clin Chem Lab Med* **2012**, *50*, 1253–1262, doi:10.1515/CCLM.2011.817.
2. Mindukshev, I.; Fock, E.; Dobrylko, I.; Sudnitsyna, J.; Gambaryan, S.; Panteleev, M.A. Platelet Hemostasis Reactions at Different Temperatures Correlate with Intracellular Calcium Concentration. *Int J Mol Sci* **2022**, *23*, 10667, doi:10.3390/ijms231810667.
3. Mikhailova, D.M.; Sudnitsyna, J.; Kovgan, P.; Naida, L.; Kharazova, A.; Mindukshev, I.; Gambaryan, S. Analysis of Ferric Protoporphyrin IX Effects on Human Platelets: Hematin Is a More Potent Agonist than Hemin. *Cells* **2025**, *14*, 255, doi:10.3390/cells14040255.
4. Ferrell, J.E.; Ha, S.H. Ultrasensitivity Part II: Multisite Phosphorylation, Stoichiometric Inhibitors, and Positive Feedback. *Trends Biochem Sci* **2014**, *39*, 556–569, doi:10.1016/j.tibs.2014.09.003.
